# Incidence and prevalence of primary care antidepressant prescribing in children and young people in England, 1998–2017: A population-based cohort study

**DOI:** 10.1371/journal.pmed.1003215

**Published:** 2020-07-22

**Authors:** Ruth H. Jack, Chris Hollis, Carol Coupland, Richard Morriss, Roger David Knaggs, Debbie Butler, Andrea Cipriani, Samuele Cortese, Julia Hippisley-Cox

**Affiliations:** 1 Division of Primary Care, School of Medicine, University of Nottingham, Nottingham, United Kingdom; 2 Division of Psychiatry and Applied Psychology, School of Medicine, University of Nottingham, Nottingham, United Kingdom; 3 National Institute for Health Research (NIHR) Nottingham Biomedical Research Centre, Nottingham, United Kingdom; 4 NIHR MindTech MedTech Co-operative, Nottingham, United Kingdom; 5 NIHR Applied Research Collaboration East Midlands, Nottingham, United Kingdom; 6 School of Pharmacy, University of Nottingham, Nottingham, United Kingdom; 7 Department of Psychiatry, University of Oxford, Oxford, UK; Oxford Health NHS Foundation Trust, Warneford Hospital, Oxford, United Kingdom; 8 Centre for Innovation in Mental Health, School of Psychology, Life and Environmental Sciences, University of Southampton, Southampton, United Kingdom; 9 Clinical and Experimental Sciences (CNS and Psychiatry), Faculty of Medicine, University of Southampton, Southampton, United Kingdom; 10 Solent NHS Trust, Southampton, United Kingdom; 11 New York University Child Study Center, New York, New York, United States of America; 12 Nuffield Department of Primary Care Health Sciences, University of Oxford, Oxford, United Kingdom; Karolinska Institutet Department of Clinical Neuroscience, SWEDEN

## Abstract

**Background:**

The use of antidepressants in children and adolescents remains controversial. We examined trends over time and variation in antidepressant prescribing in children and young people in England and whether the drugs prescribed reflected UK licensing and guidelines.

**Methods and findings:**

QResearch is a primary care database containing anonymised healthcare records of over 32 million patients from more than 1,500 general practices across the UK. All eligible children and young people aged 5–17 years in 1998–2017 from QResearch were included. Incidence and prevalence rates of antidepressant prescriptions in each year were calculated overall, for 4 antidepressant classes (selective serotonin reuptake inhibitors [SSRIs], tricyclic and related antidepressants [TCAs], serotonin and norepinephrine reuptake inhibitors [SNRIs], and other antidepressants), and for individual drugs. Adjusted trends over time and differences by social deprivation, region, and ethnicity were examined using Poisson regression, taking clustering within general practitioner (GP) practices into account using multilevel modelling. Of the 4.3 million children and young people in the cohort, 49,434 (1.1%) were prescribed antidepressants for the first time during 20 million years of follow-up. Males made up 52.0% of the cohorts, but only 34.1% of those who were first prescribed an antidepressant in the study period. The largest proportion of the cohort was from London (24.4%), and whilst ethnicity information was missing for 39.5% of the cohort, of those with known ethnicity, 75.3% were White. Overall, SSRIs (62.6%) were the most commonly prescribed first antidepressant, followed by TCAs (35.7%). Incident antidepressant prescribing decreased in 5- to 11-year-olds from a peak of 0.9 in females and 1.6 in males in 1999 to less than 0.2 per 1,000 for both sexes in 2017, but incidence rates more than doubled in 12- to 17-year-olds between 2005 and 2017 to 9.7 (females) and 4.2 (males) per 1,000 person-years. The lowest prescription incidence rates were in London, and the highest were in the South East of England (excluding London) for all sex and age groups. Those living in more deprived areas were more likely to be prescribed antidepressants after adjusting for region. The strongest trend was seen in 12- to 17-year-old females (adjusted incidence rate ratio [aIRR] 1.12, 95% confidence interval [95% CI] 1.11–1.13, p < 0.001, per deprivation quintile increase). Prescribing rates were highest in White and lowest in Black adolescents (aIRR 0.32, 95% CI 0.29–0.36, p < 0.001 [females]; aIRR 0.32, 95% CI 0.27–0.38, p < 0.001 [males]). The 5 most commonly prescribed antidepressants were either licensed in the UK for use in children and young people (CYP) or included in national guidelines. Limitations of the study are that, because we did not have access to secondary care prescribing information, we may be underestimating the prevalence and misidentifying the first antidepressant prescription. We could not assess whether antidepressants were dispensed or taken.

**Conclusions:**

Our analysis provides evidence of a continuing rise of antidepressant prescribing in adolescents aged 12–17 years since 2005, driven by SSRI prescriptions, but a decrease in children aged 5–11 years. The variation in prescribing by deprivation, region, and ethnicity could represent inequities. Future research should examine whether prescribing trends and variation are due to true differences in need and risk factors, access to diagnosis or treatment, prescribing behaviour, or young people’s help-seeking behaviour.

## Abstract 2

Ruth Jack and co-workers study antidepressant prescribing in primary care for children and adolescents in England.

## Author summary

**Why was this study done?:**

- A substantial increase has been noted in the UK and other countries in the prescription of antidepressant medicines for young people, despite their benefits and safety remaining matters for debate.
- Several antidepressants are included in UK guidelines for children and young people, though not all are currently licensed for use in under 18s in the UK.
- It remains unclear whether there is variation in prescribing across different groups and if the choice of medicines used in real-world practice adheres to evidence-based clinical guidelines.

**What did the researchers do and find?:**

- We examined the incidence and prevalence of antidepressant prescribing in a cohort of over 4.3 million 5- to 17-year-olds in England from a large primary care database between 1998 and 2017.
- Antidepressant prescribing decreased in 5- to 11-year-olds between 1999 and 2017 but more than doubled in 12- to 17-year-olds between 2005 and 2017.
- There was variation in prescribing by deprivation, region, and ethnicity, even after taking other factors into account.

**What do these findings mean?:**

- The variation in prescribing by deprivation, region, and ethnicity found could represent inequities in care and service provision.
- Future research should examine whether prescribing trends and variation are due to differences in need or barriers in accessing diagnosis or treatment.

## Introduction

Depressive disorders were the third largest cause of adolescent disability-adjusted life years lost globally in 2015 [1]. Compared with adults, children and young people (CYP) with major depressive disorder are still underdiagnosed and undertreated [2,3]. Consequences of depressive episodes in young people include serious impairments in social functioning and school performance, as well as suicidal ideation and attempts [4]. Psychological treatments are still considered the first-line treatment in many clinical guidelines, including the UK National Institute for Health and Care Excellence (NICE) guidelines for depression in CYP [5]. However, 22.7% of CYP with emotional disorders reported waiting more than 6 months to see a mental health specialist in England in 2017 [6], and antidepressants are widely used in the treatment of depression in children and adolescents.

The efficacy and safety of antidepressant medicines for major depression in CYP remains controversial [7]. Fluoxetine is the only antidepressant licensed for use in CYP as a first-line treatment for major depression in the UK [8] and the US [9]. In the UK, other antidepressants are licensed for obsessive-compulsive disorder (fluvoxamine and sertraline) and nocturnal enuresis (imipramine), and these drugs are recommended as the first antidepressants to use by the relevant NICE guidelines [10,11]. Amitriptyline has neuropathic pain listed as an unlicensed indication in the British National Formulary for Children [8] and is suggested as a prophylactic treatment for migraines in NICE guidelines on headaches in over-12s [12]. Tricyclic antidepressants, in particular imipramine, have historically been used as a second or third line drug in the management of attention-deficit hyperactivity disorder (ADHD) in children despite being unlicensed for this indication [8]. Therefore, examining the trends in antidepressants prescribed to CYP may indicate changes in relation to the indications these drugs are licensed for, as well as whether NICE guidelines and evidence-based practice are being followed. According to NICE guidelines, in the UK, prescribing antidepressants to CYP should only be done after assessment and diagnosis by a child and adolescent psychiatrist [5,10] or other specialist with expertise in child and adolescent mental health [11]. Referral to specialists in secondary care would usually be made, when appropriate, after visiting a general practitioner (GP) in primary care [13]. Work examining indications recorded in primary care around the time of the first antidepressant prescription and which secondary care specialists were seen has been done separately [14].

Antidepressant prescriptions for young people were increasing until a drop in 2002, and then began significantly increasing again from 2005 both in the UK [15–19] and other countries [20–23]. Increased prescribing of antidepressants could indicate a greater awareness and recognition of mental health and related issues and a willingness to seek help in the form of being diagnosed and/or treated. There is some evidence that in people of all ages, antidepressant prescribing varies by region [24] and between different ethnic groups in England [25,26]. Previous studies showing that CYP living in more deprived areas are more likely to receive antidepressant prescriptions have not taken region or ethnicity into account [15,16]. There is generally a lack of evidence about variation in antidepressant prescribing in CYP, particularly taking other possibly confounding factors into account. Differences in prescribing by deprivation, geographical location, and ethnicity could indicate differences in the distribution of risk factors for mental health disorders and/or differences in access to psychological therapies, which could both affect the likelihood of antidepressant prescribing.

It remains unclear, based on previous studies, whether the reported rise in antidepressant prescribing in CYP that began in 2005 is continuing and whether there is variation in prescribing across different groups. Our study aimed to examine changes over time and the variation in the use of antidepressant medicines in CYP aged between 5 and 17 years old between 1998 and 2017 in England. Our objectives were to 1) describe differences in antidepressant prescriptions for CYP over time by age, sex, deprivation, region, and ethnicity; 2) estimate variation in prescribing between these groups adjusting for the other factors; and 3) assess to what extent NICE guidelines for CYP are being adhered to.

## Methods

The full protocol for the study has previously been published [27]. Any analyses that were not prespecified in the protocol are described as sensitivity or post hoc analyses below. This study is reported as per the Strengthening the Reporting of Observational Studies in Epidemiology (STROBE) guideline (see S1 STROBE Checklist).

### Data sources

The cohort was extracted from a large primary care database (QResearch, version 43) linked to hospital episode statistics (HES) admitted patient care and outpatient data. At the time of the study, the QResearch database included health records of over 32 million patients from more than 1,500 general practices across the UK that record data using the Egton Medical Information Systems (EMIS) medical records computer system.

### Study participants

The study’s open cohort was defined as all people registered on the QResearch database in England who were aged between 5 and 17 years between 1 January 1998 and 31 December 2017. Each person’s study entry date was defined as the latest date of the following: 12 months after their registration with a study practice, 12 months after the installation date of their practice’s EMIS computer system, 1 January of the year they turned 5 years old, or 1 January 1998. People were then followed up until the earliest date of them leaving the practice, dying, 1 January of the year they turned 18 years old, or the end of the follow-up period (31 December 2017).

### Outcomes

We extracted information on prescriptions for any antidepressant for each person in the cohort. In some cases, we had prescribing information from before a patient registered with a practice because of electronic transferring of prescription data or before the practice installed EMIS. In this way, we could identify prescriptions that took place before the study period. We examined all antidepressants combined and 4 different drug classes: selective serotonin reuptake inhibitors (SSRIs), tricyclic and related antidepressants (TCAs), serotonin and norepinephrine reuptake inhibitors (SNRIs), and other antidepressants, including monoamine oxidase inhibitors (MAOIs). Antidepressants included in each drug class can be found in S1 Table. We also considered individual antidepressant drugs separately. For the figures, the 10 most prescribed antidepressants overall (each representing at least 0.8% of prescriptions) were included. Any antidepressant prescribed within the study period was included in the prevalence analyses. Those with a record of an antidepressant prescription before their study entry were excluded for the incidence cohort so that only the first antidepressant prescription was examined.

### Covariates

We analysed 4 groups, defined by sex (female and male) and age (5–11 and 12–17 years). These age groups are similar to those specified in the NICE guidelines on depression in CYP [5] but exclude 18-year-olds, who may have been treated as adults. We also studied trends and variation for deprivation, different regions of England, and ethnic groups. Deprivation was measured using the Townsend deprivation index, an area-based measure of deprivation that combines information on 4 indicators (unemployment, non-car ownership, non-home ownership, household overcrowding) from the census [28]. Areas are then divided into quintiles based on their score. When ethnicity information was missing in QResearch, we supplemented this with the most recent valid ethnic code available in HES. We examined 5 broad ethnic groups: White, Mixed, Asian, Black, and Chinese or other ethnic group, plus those with no recorded ethnicity.

### Statistical analysis

We calculated incidence rates for being first prescribed an antidepressant and prevalence rates for people with a first or subsequent antidepressant prescription per 1,000 person-years for each year between 1998 and 2017. These were produced for the different sex and age groups for all antidepressants, the drug classes, and individual drugs as described above.

Incidence rate ratios were calculated using multilevel mixed-effects Poisson regression to take account of any clustering within GP practices for the different sex and age groups. The fully adjusted models included year, region, Townsend deprivation quintile, and ethnic group. In order to take account of varying patterns in antidepressant prescribing over time, linear trends were assessed for different periods using piecewise linear regression [29] with change points identified by previous studies: 2002 and 2005 [15,17]. The year 2008 was also identified as a change point for antidepressant prevalence in people aged 14 years and over, but not incidence [17]. We included this and assessed its statistical significance because our study has a longer follow-up than the previous analysis. Sensitivity analyses recoding the not known ethnic group to White and examining prescribing by deprivation after excluding London data were also performed after examining the initial results.

As a post hoc analysis, we assessed whether the incidence rate ratios (IRRs) for 12- to 17-year-olds from the Poisson regression were linked to other factors in each region. For this, we plotted the IRRs alongside previously published data from other sources: local authority spending on ‘low-level’ mental health services (those that are nonspecialist, preventive, and early intervention, which fall below specialist referral thresholds) per child in the 2018–2019 financial year in regions in England [30] and prevalence estimates of any depressive disorder and any anxiety disorder for each region from a national survey in England in 2017 of CYP aged 5–19 years [6].

All analyses were performed using Stata/SE v15 (StataCorp LLC, TX, USA).

### Ethics statement

The project was independently peer reviewed and accepted by the QResearch Scientific board and approved in accordance with the procedure agreed with the Trent Research Ethics Committee (reference: 18/EM/0400).

## Results

**Fig 1:**
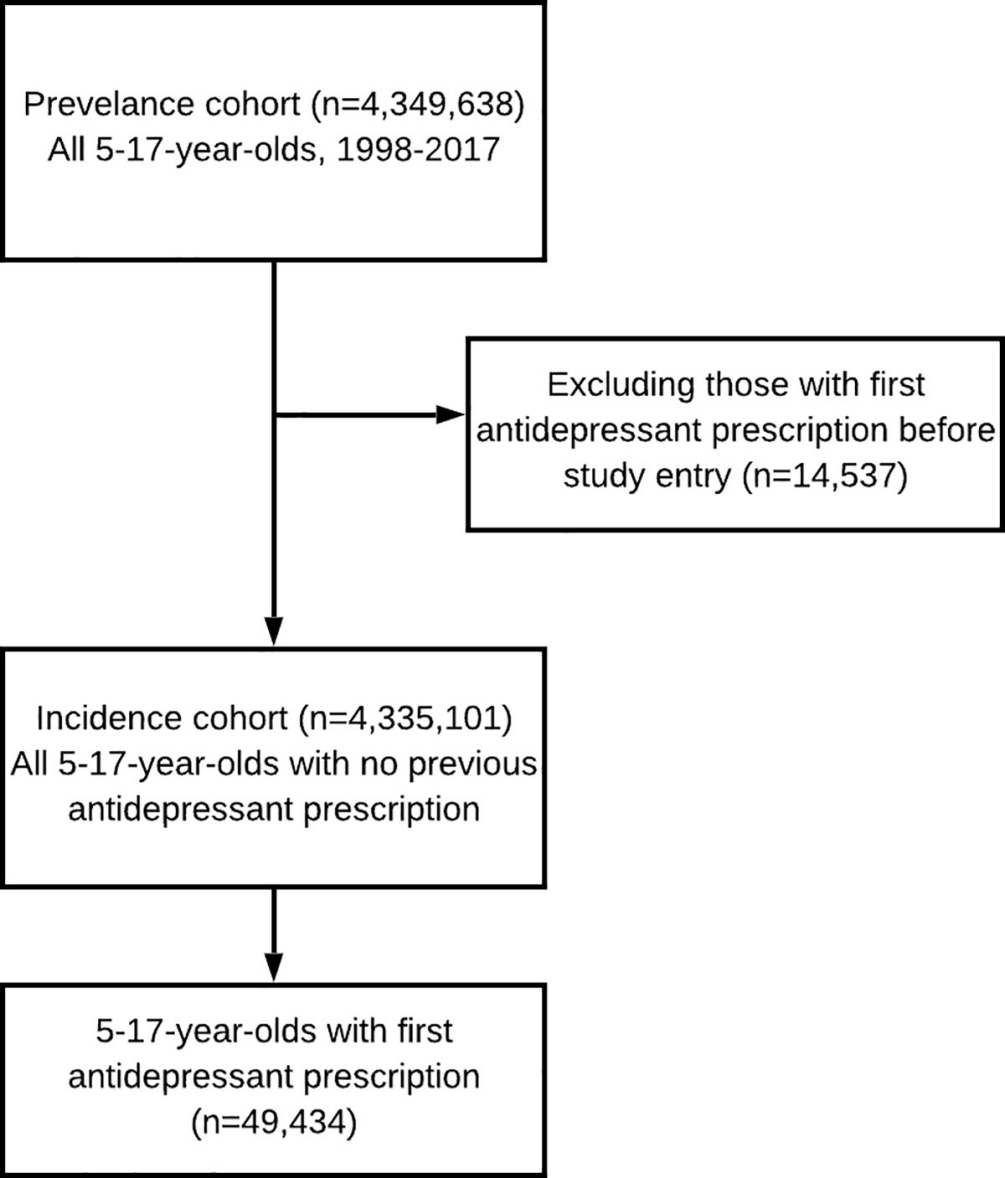
Flowchart of selection of study participants.

The flow chart detailing the selection of the cohort is shown in Fig 1. There were 4,349,638 CYP included in the prevalence study cohort. Of these, 14,537 (0.3%) had their first antidepressant prescription before their study entry date and were excluded from the incidence cohort. This left 49,434 CYP who were first prescribed an antidepressant during the study period. Details of characteristics of the incidence cohort and the subcohort prescribed their first antidepressant are shown in Table 1. Almost three-quarters of the incidence cohort (nearly 3.2 million participants) entered the study aged between 5 to 11 years, and the median follow-up time was 3.6 years (interquartile range: 1.5–7.2 years). Ethnicity information was available in QResearch for 2,317,010 (53.5%) of the incidence cohort and was supplemented by HES records for a further 307,810 (7.1%).

**Table 1:** Characteristics of incidence cohort (incidence cohort excludes 14,537 patients with first antidepressant prescription before study entry) and the subset with a new prescription of any antidepressant during the study period, age 5 to 17 years, England 1998–2017.

		Incidence Cohort		Any New Antidepressant Prescribed Subcohort	
		n	%	n	%
Total		4,335,101	100%	49,434	100%
Age*	5–11 years	11.4 m person-years		5,133	10.4%
	12–17 years	9.0 m person-years		44,301	89.6%
Sex	Female	2,080,843	48.0%	32,571	65.9%
	Male	2,254,258	52.0%	16,863	34.1%
Townsend deprivation quintile	1 (least deprived)	973,529	22.5%	11,943	24.2%
	2	923,863	21.3%	12,066	24.4%
	3	863,260	19.9%	10,856	22.0%
	4	800,643	18.5%	8,778	17.8%
	5 (most deprived)	761,722	17.6%	5,687	11.5%
	Not known	12,079	0.3%	104	0.2%
Region	East Midlands	206,792	4.8%	2,816	5.7%
	East of England	252,108	5.8%	3,807	7.7%
	London	1,056,707	24.4%	5,723	11.6%
	North East	148,885	3.4%	1,943	3.9%
	North West	672,537	15.5%	7,973	16.1%
	South East	887,291	20.5%	13,261	26.8%
	South West	430,169	9.9%	5,866	11.9%
	West Midlands	474,983	11.0%	5,464	11.1%
	Yorkshire & Humber	205,629	4.7%	2,581	5.2%
Ethnicity	Not known	1,710,263	39.5%	15,853	32.1%
	Known	2,624,838	60.5%	33,581	67.9%
Ethnic group (% of known)	White	1,975,726	75.3%	30,731	91.5%
	Mixed	86,678	3.3%	566	1.7%
	Asian	302,499	11.5%	1,419	4.2%
	Black	185,606	7.1%	562	1.7%
	Chinese/Other	74,329	2.8%	303	0.9%

*Number of person-years included given for incidence cohort by age, as some people were included in both age groups over the study period

Overall, SSRIs (62.6%) were the most commonly prescribed first antidepressant, followed by TCAs (35.7%) (Table 2). SNRIs (0.5%) and other antidepressants (1.3%) were rarely prescribed as the first antidepressant. The 5 most commonly prescribed first antidepressants were all either licensed for use in CYP (fluoxetine, imipramine, and sertraline) or mentioned in NICE guidelines (amitriptyline and citalopram, although citalopram is only recommended as a second-line antidepressant in the treatment of depression). Over a third of first prescriptions were fluoxetine, a quarter were amitriptyline, and there were similar proportions of citalopram (11.8%) and sertraline (11.4%) prescriptions. Despite being licensed for treating obsessive-compulsive disorder in CYP, fluvoxamine was rarely prescribed as a first antidepressant in the study period, making up only 0.1% of new prescriptions.

**Table 2:** Number and percentage of CYP with first prescriptions in each drug class and for individual drugs during the study period, England 1998–2017, by age and sex.

	Total		5–11 Years				12–17 Years			
			Females		Males		Females		Males	
	n	%	n	%	n	%	n	%	n	%
Any antidepressant	49,434	100%	1,845	100%	3,288	100%	30,726	100%	13,575	100%
Drug class										
SSRI	30,949	62.6%	298	16.2%	594	18.1%	20,895	68.0%	9,162	67.5%
TCA	17,624	35.7%	1,538	83.4%	2,674	81.3%	9,285	30.2%	4,127	30.4%
SNRI	263	0.5%	6	0.3%	11	0.3%	161	0.5%	85	0.6%
Other	640	1.3%	<5	<0.3%	9	0.3%	414	1.3%	214	1.6%
Individual drug										
Fluoxetine	17,493	35.4%	171	9.3%	335	10.2%	11,940	38.9%	5,047	37.2%
Amitriptyline	12,181	24.6%	709	38.4%	921	28.0%	7,627	24.8%	2,924	21.5%
Citalopram	5,828	11.8%	29	1.6%	43	1.3%	4,244	13.8%	1,512	11.1%
Sertraline	5,612	11.4%	83	4.5%	164	5.0%	3,404	11.1%	1,961	14.4%
Imipramine	3,323	6.7%	718	38.9%	1,564	47.6%	424	1.4%	617	4.5%
Paroxetine	1,535	3.1%	12	0.7%	45	1.4%	985	3.2%	493	3.6%
Dosulepin	734	1.5%	5	0.3%	6	0.2%	490	1.6%	233	1.7%
Escitalopram	472	1.0%	<5	<0.3%	<5	<0.3%	336	1.1%	133	1.0%
Nortriptyline	420	0.8%	19	1.0%	20	0.6%	290	0.9%	91	0.7%
Mirtazapine	404	0.8%	<5	<0.3%	6	0.2%	245	0.8%	151	1.1%
Lofepramine	289	0.6%	<5	<0.3%	7	0.2%	216	0.7%	62	0.5%
Venlafaxine	225	0.5%	<5	<0.3%	10	0.3%	133	0.4%	78	0.6%
Trazodone	165	0.3%	<5	<0.3%	23	0.7%	79	0.3%	60	0.4%
Fluvoxamine	61	0.1%	<5	<0.3%	<5	<0.3%	23	0.1%	31	0.2%
Duloxetine	38	0.1%	<5	<0.3%	<5	<0.3%	28	0.1%	7	0.1%

**Abbreviations:** CYP, children and young people; SNRI, serotonin and norepinephrine reuptake inhibitor; SSRI, selective serotonin reuptake inhibitor; TCA, tricyclic and related antidepressant.

TCAs accounted for over 80% of first antidepressant prescriptions in 5- to 11-year-olds, and imipramine and amitriptyline were the most commonly first-prescribed individual drugs in this age group. In adolescents, two-thirds of the newly prescribed antidepressants were SSRIs. Over the whole study period, fluoxetine (38.3%) and amitriptyline (23.8%) were the most commonly first-prescribed individual drugs in 12- to 17-year-olds.

### Incidence rates

Incidence rates for antidepressant prescribing showed distinct patterns in the age groups and were highest in 12- to 17-year-old females (S1 Fig).

**Fig 2:**
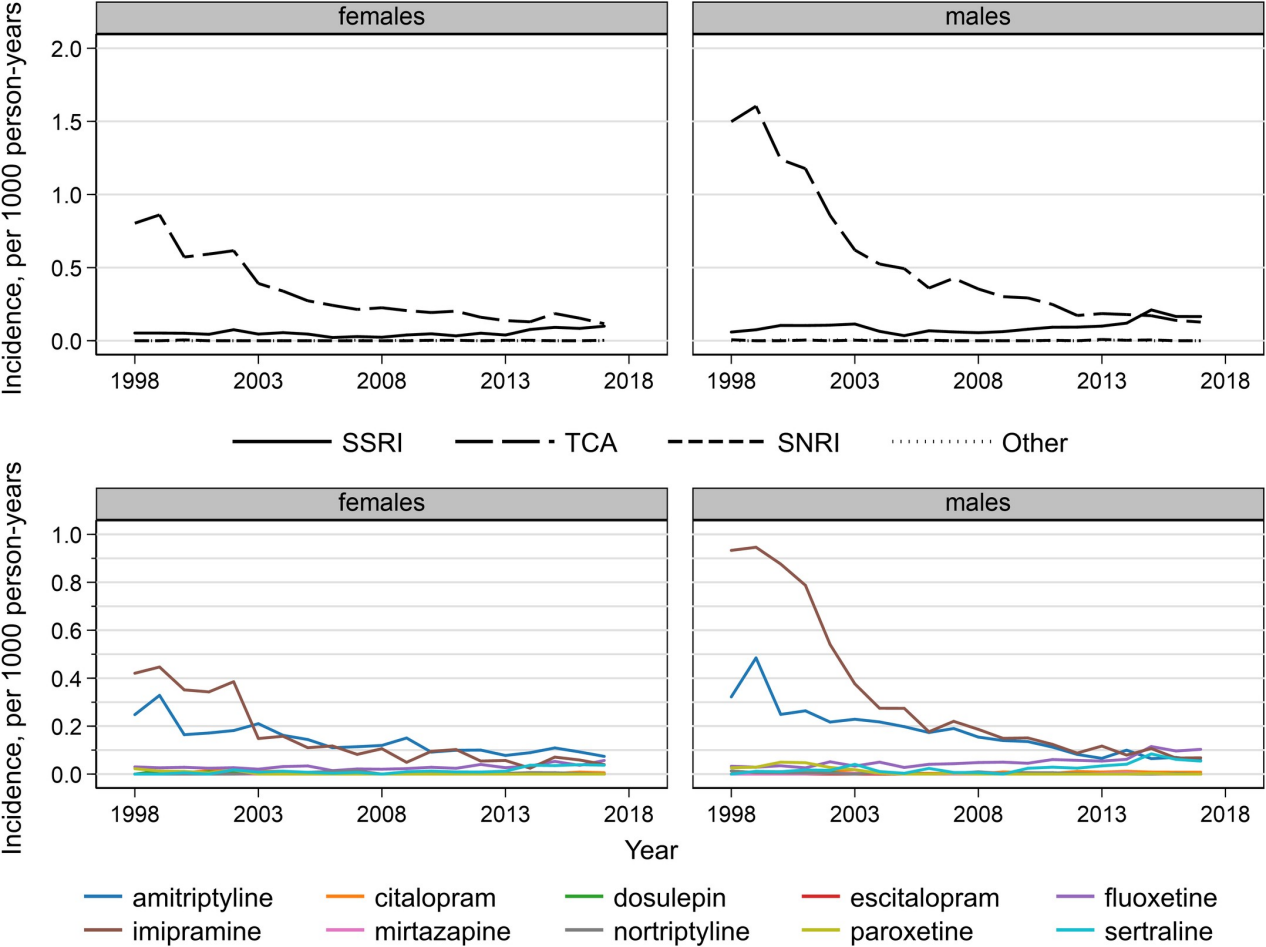
Antidepressant drug class and individual drug incidence rates per 1,000 person-years in 5- to 11-year-olds, England, 1998–2017, by sex. SNRI, serotonin and norepinephrine reuptake inhibitor; SSRI, selective serotonin reuptake inhibitor; TCA, tricyclic and related antidepressant

Antidepressant prescriptions decreased over the study period in 5- to 11-year-olds. For TCAs, incidence rates decreased by 92% from a peak of 1.6 per 1,000 person-years in males and by 86% from 0.9 in females in 1999 to less than 0.14 per 1,000 for both sexes in 2017 (Fig 2). SSRI incidence rates, however, increased from 0.05 per 1,000 person-years in females and 0.06 in males in 1998 to 0.10 in females and 0.17 in males in 2017. Imipramine was initially the most commonly prescribed individual drug for 5- to 11-year-olds. These rates decreased and were similar to amitriptyline from 2003 in females and 2006 in males. Fluoxetine and sertraline incidence rates increased over the study period so that these 4 drugs had similar incidence rates in 2017 (0.04–0.07 per 1,000 person-years in females and 0.05–0.10 in males).

**Fig 3:**
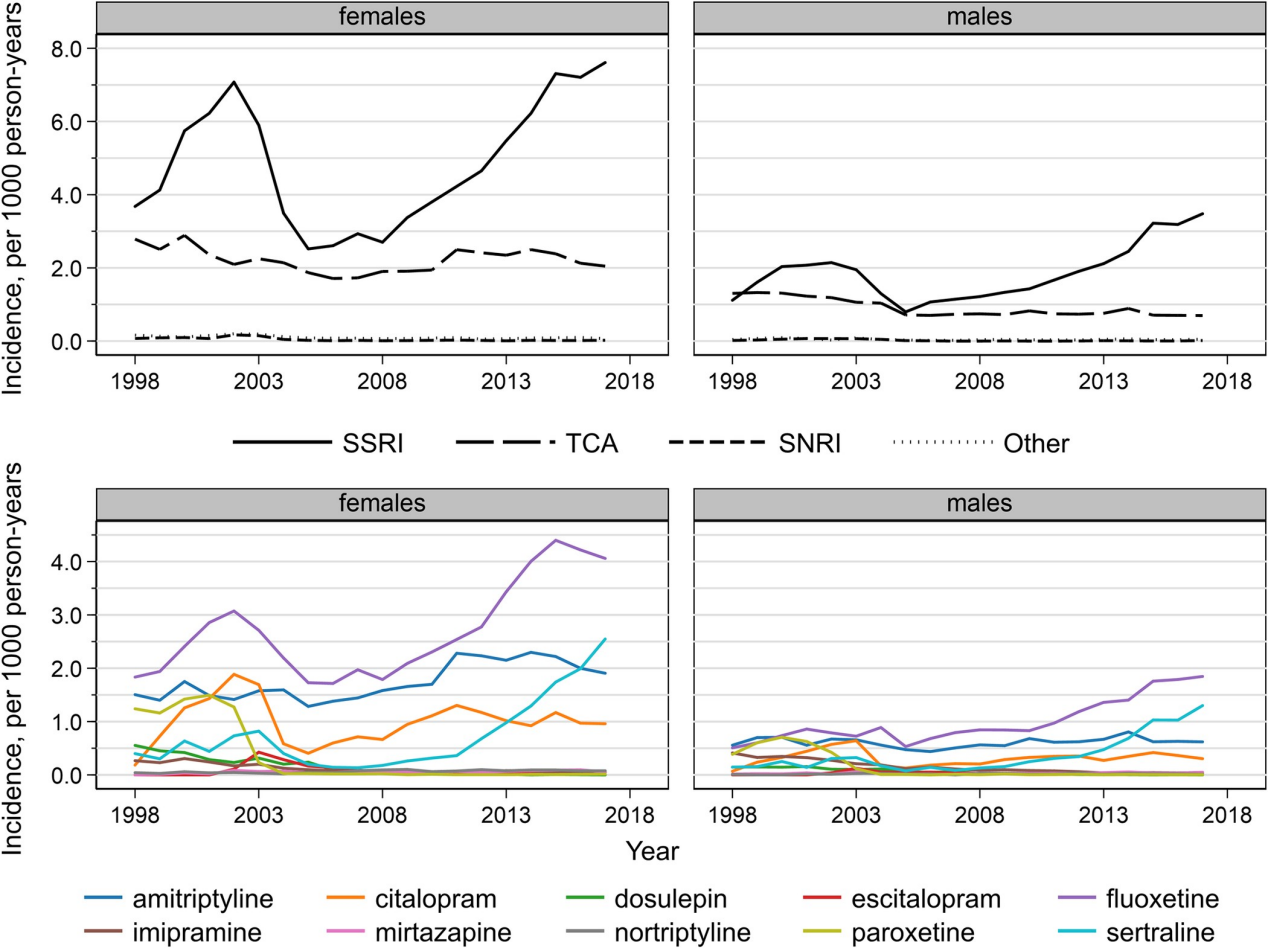
Antidepressant drug class and individual drug incidence rates per 1,000 person-years in 12- to 17-year-olds, England 1998–2017, by sex. SNRI, serotonin and norepinephrine reuptake inhibitor; SSRI, selective serotonin reuptake inhibitor; TCA, tricyclic and related antidepressant

For 12- to 17-year-olds, antidepressant incidence rates were 2.2 and 2.7 times higher in 2017 than 2005 in females and males, respectively (S1 Fig). TCA incidence rates declined over the study period to 2.0 per 1,000 person-years in females and 0.7 in males in 2017 (Fig 3). SSRI incidence rates increased between 1998 and 2002, decreased until 2005, and then increased again after this reaching a rate of 3.5 per 1,000 in males and 7.6 per 1,000 in females in 2017. The SSRI incidence rates per 1,000 person-years in 1998, 2002, and 2005 were 3.7, 7.1, and 2.5 in females and 1.1, 2.1, and 0.8 in males. Fluoxetine, sertraline, amitriptyline, and citalopram have been the 4 most commonly prescribed first antidepressants in 12- to 17-year-olds since 2003 in females and 2008 in males. This is due to a sharp decrease in paroxetine prescribing after 2002 and decreasing imipramine prescribing. The largest absolute increases were in fluoxetine and sertraline prescriptions in males and females, with sertraline becoming the second most commonly first-prescribed antidepressant for males in 2015 and for females in 2017.

S2–S4 Figs show the crude antidepressant incidence rates over time for 12- to 17-year-olds by deprivation, region, and ethnicity. These rates were lower for those in the most deprived group (S2 Fig) and in London than in other areas (S3 Fig). White males and females had the highest crude incidence rates and Black and Asian groups the lowest (S4 Fig).

### IRRs

Fully adjusted IRRs for antidepressant prescribing are shown in Table 3. In 5- to 11-year-olds, the incidence decreased throughout the time period, with the largest decrease per year between 2002 and 2005. For 12- to 17-year-olds, there was an increase in incidence rates per year from 1998 until 2002, then a decrease until 2005, a small increase until 2008, and then another increasing trend per year similar in magnitude to the first period.

**Table 3:** IRRs for any antidepressant by age and sex fully adjusted for all variables shown and accounting for clustering by GP practice, England 1998–2017.

		5–11 Years						12–17 Years					
		Females			Males			Females			Males		
		IRR	95% CI	p	IRR	95% CI	p	IRR	95% CI	p	IRR	95% CI	p
Trends within periods (per year)	1998–2002	0.92	(0.87–0.97)	0.001	0.87	(0.84–0.90)	<0.001	1.10	(1.08–1.12)	<0.001	1.08	(1.05–1.10)	<0.001
	2002–2005	0.78	(0.73–0.84)	<0.001	0.78	(0.74–0.82)	<0.001	0.76	(0.74–0.77)	<0.001	0.77	(0.74–0.79)	<0.001
	2005–2008	0.92	(0.85–0.98)	0.015	0.91	(0.86–0.96)	<0.001	1.03	(1.01–1.05)	0.002	1.01	(0.98–1.04)	0.397
	2008–2017	0.97	(0.95–1.00)	0.036	0.95	(0.94–0.97)	<0.001	1.09	(1.08–1.09)	<0.001	1.08	(1.08–1.09)	<0.001
Townsend deprivation quintile	Q1 (least deprived)	1.00			1.00			1.00			1.00		
	Q2	1.09	(0.95–1.24)	0.226	0.96	(0.87–1.06)	0.432	1.15	(1.11–1.18)	<0.001	1.14	(1.09–1.20)	<0.001
	Q3	1.09	(0.95–1.26)	0.223	1.11	(1.00–1.23)	0.053	1.30	(1.26–1.35)	<0.001	1.23	(1.17–1.30)	<0.001
	Q4	1.25	(1.08–1.46)	0.004	1.18	(1.05–1.32)	0.005	1.46	(1.40–1.52)	<0.001	1.37	(1.29–1.45)	<0.001
	Q5 (most deprived)	1.33	(1.11–1.59)	0.002	1.13	(0.98–1.30)	0.089	1.48	(1.41–1.55)	<0.001	1.29	(1.20–1.39)	<0.001
	Not known	1.51	(0.67–3.39)	0.315	1.27	(0.66–2.45)	0.480	1.12	(0.88–1.44)	0.349	1.18	(0.79–1.76)	0.428
	Deprivation trend (excluding not known)	1.07	(1.03–1.12)	0.001	1.05	(1.02–1.08)	0.002	1.12	(1.11–1.13)	<0.001	1.08	(1.07–1.10)	<0.001
Region	East Midlands	1.00			1.00			1.00			1.00		
	East of England	0.71	(0.49–1.04)	0.077	0.77	(0.56–1.07)	0.116	1.18	(1.02–1.36)	0.027	1.12	(0.95–1.32)	0.191
	London	0.50	(0.37–0.69)	<0.001	0.48	(0.37–0.63)	<0.001	0.44	(0.39–0.49)	<0.001	0.52	(0.45–0.60)	<0.001
	North East	0.93	(0.61–1.40)	0.716	0.86	(0.60–1.24)	0.422	0.76	(0.64–0.89)	0.001	0.75	(0.62–0.91)	0.004
	North West	0.74	(0.54–1.01)	0.062	0.66	(0.50–0.87)	0.003	0.81	(0.72–0.91)	0.001	0.90	(0.78–1.04)	0.153
	South East	1.03	(0.76–1.39)	0.861	1.08	(0.83–1.40)	0.570	1.20	(1.06–1.35)	0.003	1.29	(1.12–1.48)	<0.001
	South West	0.87	(0.63–1.22)	0.422	0.83	(0.62–1.11)	0.205	1.10	(0.96–1.25)	0.162	1.02	(0.87–1.19)	0.827
	West Midlands	0.80	(0.57–1.11)	0.174	0.82	(0.62–1.09)	0.173	0.86	(0.76–0.98)	0.024	0.94	(0.81–1.09)	0.433
	Yorkshire & Humber	0.99	(0.68–1.45)	0.971	0.98	(0.71–1.36)	0.913	0.81	(0.69–0.94)	0.006	0.77	(0.65–0.93)	0.006
Ethnic group	White	1.00			1.00			1.00			1.00		
	Mixed	0.75	(0.50–1.15)	0.187	0.85	(0.62–1.17)	0.319	0.66	(0.59–0.74)	<0.001	0.81	(0.70–0.94)	0.007
	Asian	0.70	(0.54–0.89)	0.004	0.64	(0.52–0.78)	<0.001	0.41	(0.38–0.44)	<0.001	0.47	(0.42–0.52)	<0.001
	Black	0.71	(0.51–0.98)	0.038	0.56	(0.42–0.75)	<0.001	0.32	(0.29–0.36)	<0.001	0.32	(0.27–0.38)	<0.001
	Chinese/Other	0.49	(0.26–0.91)	0.025	0.47	(0.28–0.79)	0.004	0.44	(0.38–0.51)	<0.001	0.57	(0.47–0.69)	<0.001
	Not known	0.52	(0.47–0.58)	<0.001	0.54	(0.49–0.58)	<0.001	0.45	(0.44–0.46)	<0.001	0.41	(0.39–0.42)	<0.001

**Abbreviations:** CI, confidence interval; GP, general practitioner; IRR, incidence rate ratio.

Antidepressant incidence rates increased with increasing deprivation in all 4 age–sex subgroups after adjustment. The strongest trend was seen in 12- to 17-year-old females (adjusted IRR = 1.12, 95% confidence interval [CI] 1.11–1.13, p < 0.001, per deprivation quintile increase). However, unadjusted estimates of antidepressant prescribing by deprivation showed a statistically significant trend of decreasing prescribing with increasing deprivation, driven by the most deprived quintile, in all age and sex groups apart from 5- to 11-year-old females (S3 Table). For the older age group, adjusting for region resulted in increased prescribing rates with increasing deprivation, whereas adjusting for year alone did not affect the unadjusted trend results, and adjusting for just ethnicity attenuated the association but showed those living in more deprived areas were still less likely to be prescribed antidepressants. This statistically significant pattern with deprivation was also shown for 5- to 11-year-old females after adjusting for region. This association was only evident after taking GP practice clustering into account in the fully adjusted models (Table 3) in 5- to 11-year-old males. Within London, there was a statistically significant unadjusted association with deprivation and prescribing in 12- to 17-year-olds, with those living in more deprived areas having lower prescribing rates. After excluding London in the unadjusted analyses, increased prescribing with increasing deprivation was found for all groups apart from 5- to 11-year-old males, highlighting the influence of region on these results.

London had the lowest IRRs and the South East had the highest IRRs in all sex and age subgroups. The South East estimates were all more than double that of London. The North East had the second lowest prescribing rate estimates for 12- to 17-year-olds. These estimates were 1.4 (in males) and 1.7 (in females) times higher than the London estimates.

Asian, Black, and Chinese or other ethnic groups, as well as those whose ethnicity was not known, had statistically significantly lower prescription incidence rates compared with the White baseline group in 5- to 11-year-olds. For the older age group, the Mixed ethnic group also had significantly lower incidence rates than the White group. In both males and females, the lowest IRRs in adolescents were found in the Black group (IRR 0.32, 95% CI 0.27–0.38, p < 0.001 and IRR 0.32, 95% CI 0.29–0.36, p < 0.001, respectively) compared with the White group. Results were attenuated in the sensitivity analysis in which the not known ethnic group were recoded as White (S2 Table). There were no statistically significant differences between all other ethnic groups and the White/not known baseline for females aged 5–11 years. In males aged 5–11 years in the Chinese or other (IRR 0.59, 95% CI 0.36–0.99, p = 0.046), Black (IRR 0.72, 95% CI 0.53–0.96, p = 0.027), and Asian (IRR 0.83, 95% CI 0.68–1.01, p = 0.067) ethnic groups, the IRRs were still lower than the White/not known group, although the Asian group was only borderline statistical significance. In the older age group, the attenuated results showed the same patterns, with only the male Mixed ethnic group no longer statistically significantly different from the baseline.

### Prevalence rates

The prevalence rate patterns overall, in antidepressant drug classes, and in individual drugs were similar to the incidence rates (S1, S5 and S6 Figs). Prevalence estimates for any antidepressant prescriptions were highest for 5- to 11-year-olds in 1999 and decreased over the study period. Despite a peak in the early 2000s in prescriptions for 12- to 17-year-olds, the highest rates for males and females were in 2017 (7.9 and 16.4 per 1,000 person-years, respectively). In 12- to 17-year-olds, there were an extra 6.6 females and 3.7 males per 1,000 person-years who had a second or later antidepressant prescription in 2017. There were very small differences between the incidence and prevalence rates in 5- to 11-year-olds throughout the study period.

S7 Fig shows a negative association between spending per child on ‘low-level’ mental health services and the prescribing patterns from our study. There appears to be no strong correlation between either spending or prescribing and the depression and anxiety prevalence estimates.

## Discussion

### Summary of main results

This study has shown a diverging pattern of decreasing prescribing of antidepressants in 5- to 11-year-olds between 1998 and 2017, whilst rates have more than doubled in 12- to 17-year-olds since 2005. The most commonly first-prescribed antidepressants are either licensed for use in CYP or included in national guidelines. Rates of antidepressant prescribing were higher for CYP living in more deprived areas after accounting for GP practice clustering and region. Children and adolescents living in South East England were more likely to be prescribed antidepressants, and those living in London the least likely. Prescribing rates were highest in White and lowest in Black adolescents.

### Comparison with other studies

This study has shown that the increase in antidepressant prescribing in CYP in England from 2005 previously found [15–19] has continued until 2017. The 2017 antidepressant prescribing prevalence rates in adolescents from our study (0.79% and 1.64%) are around half the associated depressive disorder estimates (1.6% and 3.8%) [6] in males and females, respectively. Citalopram prescribing has previously been found to be higher than in our study—higher than fluoxetine in 6- to 18-year-olds [16], similar to fluoxetine in under-18s in 2009 [15], and the second most commonly prescribed antidepressant after fluoxetine in people aged under 20 [20]. Whilst the inclusion of those aged 18 years [16] and over [20] who may have been treated as adults may explain the higher rates, other methodological differences, such as variation in factors not accounted for in the unadjusted analyses, could also be important.

A previous study reported that for people of all ages in England, the prevalence of antidepressant prescriptions was lowest in London and highest in the North East of England [24]. We also found the lowest incident prescription rates in London; however, the North East was the next lowest area for adolescents. There could be differences in antidepressant prescribing patterns between CYP and adults, including initiating prescribing and the number of prescriptions because of the length of time people stay on antidepressants affecting the prevalence analysis, that account for this difference. Our study examined prescriptions, whilst Grigoroglou and colleagues [24] used dispensing data, so differences in whether patients filled their prescriptions may also exist.

A report by the Children’s Commissioner into early access to mental health support found variation in the stated spending on ‘low-level’ preventive and early-intervention (including nonspecialist psychological support services) services between different regions [30]. London had the highest spending, and the East of England spent the least per child. We found that the lowest prescribing for adolescents was in London, which is located between the South East and East of England regions. These 2 areas had the highest prescribing IRR estimates for 12- to 17-year-olds. S7 Fig shows there appears to be a negative association between spending per child on ‘low-level’ mental health services (London highest; East of England and South East lowest) and the prescribing patterns from our study (London lowest; East of England and South East highest). This implies that antidepressants might be prescribed more frequently to CYP in areas where there is less investment in preventive services or psychological support available, although further research is needed to test this hypothesis. That there was no strong correlation between either spending or prescribing and the depression and anxiety prevalence estimates could be due to differences in age groups and years studied.

Our study has good face validity because we found similar trends to those published elsewhere. For example, we found that after adjustment for GP practice clustering and region, CYP living in more deprived areas are more likely to receive antidepressant prescriptions compared with those in the less deprived areas, which has previously been shown for children and adolescents [15,16] and adults [24] in the UK. Although the unadjusted results showed lower antidepressant prescribing in adolescents living in the most deprived quintile (S2 Fig), this was due to the lower prescribing and large proportion of people living in the most deprived quintile in London. Excluding London from an unadjusted analysis of trend over deprivation changed the estimates in 12- to 17-year-olds from 0.96 (p < 0.001) in both sexes to 1.06 in females and 1.05 in males (both p < 0.001) (S3 Table). Measures of deprivation were not found to be statistically significantly associated with spending on CYP mental health per child at the Clinical Commissioning Group level in a study by Rocks and colleagues [31]. This study included early help and targeted services (tier 2) and specialised Child and Adolescent Mental Health Services (CAMHS) (tier 3). Spending on early interventions by nonspecialists (tier 1) could influence antidepressant prescriptions and might be linked to deprivation.

### Strengths and limitations

The main strengths of this study are its size, representativeness, and duration. We analysed data from over 4.3 million CYP across England over a 20-year period, which, to our knowledge, is the largest study to date on antidepressant prescribing in CYP. The vast majority of the UK population are registered with a GP practice [13], and the QResearch database is the most nationally representative primary care database in England [32] with comprehensive information on prescriptions. This means that the results are likely to generalise well within the UK. We have been able to examine a long time series for different sex and age groups and investigate variation by deprivation, region, and ethnicity whilst taking all these factors into account.

A limitation of the study is that it does not include any secondary care prescriptions. There is no publicly available information about antidepressant prescribing for CYP in secondary care in the UK. Most parents would initially first visit a GP with concerns about their child’s mental health issues [33]. The GP would refer them to a specialist in child and mental health following if appropriate [5,10]. Furthermore, in the UK, GPs are typically responsible for the ongoing prescribing of antidepressant medicines if initiated by a specialist. It is possible that what we have identified as a first prescription is actually a subsequent prescription in primary care following an initial prescription in secondary care. Without secondary care prescriptions, the prevalence estimates will be underestimates of the total antidepressant prescriptions in CYP. However, the prevalence patterns should be unaffected by who initiated the first prescription if prescribing is transferred to primary care. We were only able to assess whether antidepressants were prescribed, not whether they were dispensed or taken. Unmeasured confounding may also still be present. We chose to include all CYP regardless of whether they had an appropriate indication recorded and cannot be sure that particular antidepressants were prescribed for the indications they are licensed for.

Because we used data routinely recorded in primary care rather than prospectively collecting the data specifically for the study, not all information we required was available. Despite using all available ethnicity information from QResearch and HES records, ethnicity was still missing for 40%. As a sensitivity analysis, we recoded those with no ethnicity information to a new White/not known group. This attenuated the results for the other ethnic groups, but these were still statistically significant for females aged 12–17 years, males aged 12–17 years (apart from the Mixed group), and Black and Chinese or other ethnic groups in males aged 5–11 years.

### Implications

Our study was not designed to determine whether increased prescribing of antidepressants in CYP since 2005 is due to increasing rates of mental health problems, greater awareness and help-seeking for those with mental health issues, prescribing behaviour, patient choice, or because there are issues with accessing other psychological therapies [6]. Generally, the antidepressants prescribed to CYP appear to be those licensed for use in under-18s in the UK or listed for use with particular indications in the British National Formulary for Children [8] despite not being licensed. The rapid recent increase of sertraline as the first antidepressant prescribed is of interest. Sertraline is licensed in the UK for use in CYP for obsessive-compulsive disorder and recommended as a second-line treatment for major depression in CYP by NICE [5]. The prevalence of obsessive-compulsive disorder is low in England (0.4% in 2017) [6], and as a second-line drug, sertraline would not be expected to be the first antidepressant prescribed for major depression. There are currently no UK guidelines on treating CYP with anxiety, although meta-analyses have shown SSRIs, including sertraline, to be effective [34]. General practitioners may be following the adult guidance, which suggests sertraline and was published in 2011 [35], after which female adolescent sertraline prescription rates rapidly increased. The relatively high prescribing rates of amitriptyline should be explored further to determine what indications this drug is being prescribed for. Whilst amitriptyline is unlicensed for use in CYP, it is included in NICE guidelines for treating headaches [12] and the British National Formulary for Children for treating neuropathic pain [8]. TCAs in general are not recommended for depressive disorder in CYP and those who self-harm [36] because of life-threatening cardiac risks associated with overdose, but rates of self-harm have been shown to be increasing in 13- to 16-year-olds in recent years in the UK [37]. Understanding which indications are recorded around the time of these first antidepressant prescriptions is important, and work examining this has been analysed separately [14].

Future work should further examine whether any barriers in awareness, access to appropriate healthcare services, and treatment exist, and if so, whether they vary for different groups and how they can be tackled. Determining whether increased spending on preventive, early-intervention services and access to psychological therapies leads to lower antidepressant prescription rates, and how these services compare in terms of outcomes is an important area of work that could lead to possible savings in healthcare spending and improved health for the population.

## Supporting information

S1 STROBE Checklist STROBE Statement for ‘Incidence and prevalence of primary care antidepressant prescribing in children and young people in England, 1998–2017: a population-based cohort study’. (DOCX)

S1 Table Antidepressants included in each drug class. (XLSX)

S2 Table IRRs for any antidepressant for recoded ethnic groups fully adjusted for year, deprivation, and region and accounting for clustering by GP practice, England 1998–2017, by age and sex. GP, general practitioner; IRR, incidence rate ratio
(XLSX)

S3 Table Unadjusted IRRs for any antidepressant for deprivation overall, London only, and excluding London and total population adjusted separately for region, year, and ethnicity, England 1998–2017, by age and sex. IRR, incidence rate ratio
(XLSX)

S1 Fig Overall antidepressant drug incidence and prevalence rates per 1,000 person-years, England 1998–2017, by age and sex. (TIF)

S2 Fig Antidepressant group incidence rates and 95% CIs per 1,000 person-years in 12- to 17-year-olds, England 1998–2017, by Townsend deprivation quintile (excluding not known) and sex. CI, confidence interval; Townsend Q1, least deprived quintile; Townsend Q5, most deprived quintile
(TIF)

S3 Fig Antidepressant group incidence rates and 95% CIs per 1,000 person-years in 12- to 17-year-olds, England 1998–2017, by region and sex. CI, confidence interval
(TIF)

S4 Fig Antidepressant group incidence rates and 95% CIs per 1,000 person-years in 12- to 17-year-olds, England 1998–2017, by ethnic group and sex. CI, confidence interval
(TIF)

S5 Fig Antidepressant drug class and individual drug prevalence rates per 1,000 person-years in 5- to 11-year-olds, England 1998–2017, by sex. (TIF)

S6 Fig Antidepressant drug class and individual drug prevalence rates per 1,000 person-years in 12- to 17-year-olds, England 1998–2017, by sex. (TIF)

S7 Fig **Regional (A) fully adjusted IRRs for any antidepressant, age 12–17 for males and females, 1998–2017; (B) LA spending on ‘low-level’ mental health services per child, 2018–2019 [30]; (C) prevalence of any depressive disorder, age 5–19, 2017 [6]; and (D) prevalence of any anxiety disorder, age 5–19, 2017 [6].** IRR, incidence rate ratio; LA, Local Authority.
(TIF)

## Abbreviations

ADHD: attention-deficit hyperactive disorder
aIRR: adjusted IRR
CAMHS: Child and Adolescent Mental Health Services
CI: confidence interval
CYP: children and young people
EMIS: Egton Medical Information Systems
GP: general practitioner
HES: hospital episode statistics
IRR: incidence rate ratio
MAOI: monoamine oxidase inhibitor
NHS: National Health Service
NICE: National Institute for Health and Care Excellence
NIHR: National Institute for Health Research
SNRI: serotonin and norepinephrine reuptake inhibitor
SSRI: selective serotonin reuptake inhibitor
TCA: tricyclic and related antidepressant

## References

[1] World Health Organization Global Accelerated Action for the Health of Adolescents (AA-HA!): guidance to support country implementation. Geneva: World Health Organization; 2017.

[2] Soria-Saucedo R, Walter HJ, Cabral H, England MJ, Kazis LE. Receipt of Evidence-Based Pharmacotherapy and Psychotherapy Among Children and Adolescents With New Diagnoses of Depression. Psychiat Serv. 2016;67(3):316–23. 10.1176/appi.ps.201500090 26725295

[3] Department of Health and Social Care and Department of Education. Transforming Children and Young People's Mental Health Provision: a Green Paper. 2017 [cited 2020 May 11]. Available from: https://www.gov.uk/government/consultations/transforming-children-and-young-peoples-mental-health-provision-a-green-paper

[4] Hopkins K, Crosland P, Elliott N, Bewley S, Clinical Guidelines Update Committee B. Diagnosis and management of depression in children and young people: summary of updated NICE guidance. BMJ. 2015;350:h824 10.1136/bmj.h824 25739880

[5] National Institute for Health and Care Excellence. Depression in children and young people: identification and management (NICE guideline NG134). 2019 [cited 2020 May 11]. Available from: https://www.nice.org.uk/guidance/ng134 31577402

[6] Sadler K, Vizard T, Ford T, Marchesell F, Pearce N, Mandalia D, et al Mental Health of Children and Young People in England, 2017 [Internet]. 2018 [cited 2020 May 11]. Available from: https://digital.nhs.uk/data-and-information/publications/statistical/mental-health-of-children-and-young-people-in-england/2017/2017

[7] Cipriani A, Zhou X, Del Giovane C, Hetrick SE, Qin B, Whittington C, et al Comparative efficacy and tolerability of antidepressants for major depressive disorder in children and adolescents: a network meta-analysis. Lancet. 2016;388(10047):881–90. 10.1016/S0140-6736(16)30385-3 27289172

[8] Paediatric Formulary Committee. BNF for Children (online) London: BMJ Group, Pharmaceutical Press, and RCPCH Publications. 2020 [cited 2020 May 11]. Available from: https://about.medicinescomplete.com/publication/british-national-formulary-for-children/

[9] Birmaher B, Brent D, AACAP Work Group on Quality Issues, Bernet W, Bukstein O, Walter H, et al Practice parameter for the assessment and treatment of children and adolescents with depressive disorders. J Am Acad Child Adolesc Psychiatry. 2007;46(11):1503–26. 10.1097/chi.0b013e318145ae1c 18049300

[10] National Institute for Health and Care Excellence. Obsessive-compulsive disorder and body dysmorphic disorder: treatment (Clinical guideline CG31). 2005 [cited 2020 May 11]. Available from: https://www.nice.org.uk/guidance/cg31 31869034

[11] National Institute for Health and Care Excellence. Bedwetting in under 19s (Clinical guideline CG111). 2010 [cited 2020 May 11]. Available from: https://www.nice.org.uk/guidance/cg111 31846260

[12] National Institute for Health and Care Excellence. Headaches in over 12s: diagnosis and management (Clinical guideline CG150) London: National Institute for Health and Care Excellence 2012 (updated 2015) [cited 2020 May 11]. Available from: https://www.nice.org.uk/guidance/cg150

[13] Vezyridis P, Timmons S. Evolution of primary care databases in UK: a scientometric analysis of research output. BMJ Open. 2016;6(10):e012785 10.1136/bmjopen-2016-012785 27729352 PMC5073525

[14] Jack RH, Joseph RM, Coupland C, Butler D, Hollis C, Morriss R, et al Secondary care specialist visits made by children and young people prescribed antidepressants in primary care: a descriptive study using the QResearch database. BMC Medicine. 2020;18(1):93 10.1186/s12916-020-01560-7 32349753 PMC7191694

[15] Wijlaars LPMM, Nazareth I, Petersen I Trends in Depression and Antidepressant Prescribing in Children and Adolescents: A Cohort Study in The Health Improvement Network (THIN). PLoS ONE. 2012;7(3):e33181 10.1371/journal.pone.0033181 22427983 PMC3302807

[16] John A, Marchant AL, Fone DL, McGregor JI, Dennis MS, Tan JOA, et al Recent trends in primary-care antidepressant prescribing to children and young people: an e-cohort study. Psychol Med. 2016;46(16):3315–27. 10.1017/S0033291716002099 27879187 PMC5122314

[17] Mars B, Heron J, Kessler D, Davies NM, Martin RM, Thomas KH, et al Influences on antidepressant prescribing trends in the UK: 1995–2011. Soc Psychiatry Psychiatr Epidemiol. 2017;52(2):193–200. 10.1007/s00127-016-1306-4 27885400 PMC5329088

[18] Sarginson J, Webb RT, Stocks SJ, Esmail A, Garg S, Ashcroft DM. Temporal trends in antidepressant prescribing to children in UK primary care, 2000–2015. J Affect Disorders. 2017;210:312–8. 10.1016/j.jad.2016.12.047 28068620 PMC5458802

[19] Tiffin PA, Mediavilla JL, Close H, Kasim AS, Welsh P, Paton LW, et al What were the impacts of the Committee on Safety of Medicines warning and publication of the NICE guidelines on trends in child and adolescent antidepressant prescribing in primary care? A population based study. BMJ Open. 2019;9(8):e028201 10.1136/bmjopen-2018-028201 31391190 PMC6686994

[20] Bachmann CJ, Aagaard L, Burcu M, Glaeske G, Kalverdijk LJ, Petersen I, et al Trends and patterns of antidepressant use in children and adolescents from five western countries, 2005–2012. Eur Neuropsychopharm. 2016;26(3):411–9. 10.1016/j.euroneuro.2016.02.001 26970020

[21] Schröder C, Dörks M, Kollhorst B, Blenk T, Dittmann RW, Garbe E, et al Outpatient antidepressant drug use in children and adolescents in Germany between 2004 and 2011. Pharmacoepidem Dr S. 2017;26(2):170–9. 10.1002/pds.4138 27868277

[22] Revet A, Montastruc F, Raynaud JP, Baricault B, Montastruc JL, Lapeyre-Mestre M. Trends and Patterns of Antidepressant Use in French Children and Adolescents From 2009 to 2016: A Population-Based Study in the French Health Insurance Database. J Clin Psychopharm. 2018;38(4):327–35. 10.1097/JCP.0000000000000891 29851707

[23] Lagerberg T, Molero Y, D'Onofrio BM, Fernandez de la Cruz L, Lichtenstein P, Mataix-Cols D, et al Antidepressant prescription patterns and CNS polypharmacy with antidepressants among children, adolescents, and young adults: a population-based study in Sweden. Eur Child Adoles Psy. 2019;28(8):1137–45. 10.1007/s00787-018-01269-2 30659386 PMC6675912

[24] Grigoroglou C, Munford L, Webb RT, Kapur N, Ashcroft DM, Kontopantelis E. Prevalence of mental illness in primary care and its association with deprivation and social fragmentation at the small-area level in England. Psychol Med. 2020;50(2):293–302. 10.1017/S0033291719000023 30744718 PMC7083582

[25] Walters P, Ashworth M, Tylee A. Ethnic density, physical illness, social deprivation and antidepressant prescribing in primary care: ecological study. Br J Psychiatry. 2008;193(3):235–9. 10.1192/bjp.bp.107.038299 18757984

[26] Schofield P, Das-Munshi J, Mathur R, Congdon P, Hull S. Does depression diagnosis and antidepressant prescribing vary by location? Analysis of ethnic density associations using a large primary-care dataset. Psychol Med. 2016;46(6):1321–9. 10.1017/S0033291715002913 26879871 PMC4828938

[27] Jack RH, Hollis C, Coupland C, Morriss R, Knaggs RD, Cipriani A, et al Trends in antidepressant prescriptions in children and young people in England, 1998–2017: protocol of a cohort study using linked primary care and secondary care datasets. Evid Based Ment Health. 2019;22(3):129–33. 10.1136/ebmental-2019-300097 31253602 PMC6662952

[28] Townsend P. Deprivation. J Soc Policy. 1987;16:125–46. 10.1017/S0047279400020341 WOS:A1987J267500001

[29] Gould WW. Linear splines and piecewise linear functions. Stata Technical B. 1993;15:13–7.

[30] Children's Commissioner. Early access to mental health support London: Children's Commissioner for England; 2019.

[31] Rocks S, Fazel M, Tsiachristas A. Variation in spend on young mental health across Clinical Commissioning Groups in England: a cross-sectional observational study. BMJ Open. 2019;9(10):e030011 10.1136/bmjopen-2019-030011 31594880 PMC6797274

[32] Kontopantelis E, Stevens RJ, Helms PJ, Edwards D, Doran T, Ashcroft DM. Spatial distribution of clinical computer systems in primary care in England in 2016 and implications for primary care electronic medical record databases: a cross-sectional population study. BMJ Open. 2018;8(2):e020738 10.1136/bmjopen-2017-020738 29490968 PMC5855245

[33] Rainer C, Crellin R. Finding Help: Children, young people and families navigating the system to get the mental health support they need London, UK: The Children's Society; 2019.

[34] Wang Z, Whiteside SPH, Sim L, Farah W, Morrow AS, Alsawas M, et al Comparative Effectiveness and Safety of Cognitive Behavioral Therapy and Pharmacotherapy for Childhood Anxiety Disorders: A Systematic Review and Meta-analysis. JAMA Pediatr. 2017;171(11):1049–56. 10.1001/jamapediatrics.2017.3036 28859190 PMC5710373

[35] National Institute for Health and Care Excellence. Generalised anxiety disorder and panic disorder in adults: management (Clinical guideline CG113). 2011 [cited 2020 May 11]. Available from: https://www.nice.org.uk/guidance/cg113 31961629

[36] National Institute for Health and Care Excellence. Self-harm in over 8s: long-term management (Clinical guideline CG133). 2011 [cited 2020 May 11]. Available from: https://www.nice.org.uk/guidance/cg133 31891461

[37] Morgan C, Webb RT, Carr MJ, Kontopantelis E, Green J, Chew-Graham CA, et al Incidence, clinical management, and mortality risk following self harm among children and adolescents: cohort study in primary care. BMJ. 2017;359 10.1136/bmj.j4351 29046278 PMC5641980

