# Supplementary material for: Incidence and prevalence of primary care antidepressant prescribing in children and young people in England, 1998–2017: A population-based cohort study
Source: PLoS Med. 2020 Jul 22;17(7):e1003215. doi: 10.1371/journal.pmed.1003215 (PMC7375537; doi:10.1371/journal.pmed.1003215)
Supplement: S1 STROBE Checklist — (DOCX) [file pmed.1003215.s001.docx]

STROBE Statement for ‘Incidence and prevalence of primary care antidepressant prescribing in children and young people in England, 1998-2017: a population-based cohort study’

|  | Item No | Recommendation |  |
| --- | --- | --- | --- |
| **Title and abstract** | 1 | (*a*) Indicate the study’s design with a commonly used term in the title or the abstract | Title |
|  |  | (*b*) Provide in the abstract an informative and balanced summary of what was done and what was found | Abstract |
| Introduction | | |  |
| Background/rationale | 2 | Explain the scientific background and rationale for the investigation being reported | Introduction, paragraphs 1-3 |
| Objectives | 3 | State specific objectives, including any prespecified hypotheses | Introduction, paragraph 4 |
| Methods | | |  |
| Study design | 4 | Present key elements of study design early in the paper | Methods, Data sources |
| Setting | 5 | Describe the setting, locations, and relevant dates, including periods of recruitment, exposure, follow-up, and data collection | Methods, Study participants |
| Participants | 6 | (*a*) Give the eligibility criteria, and the sources and methods of selection of participants. Describe methods of follow-up | Methods, Study participants |
|  |  | (*b*) For matched studies, give matching criteria and number of exposed and unexposed | Not applicable |
| Variables | 7 | Clearly define all outcomes, exposures, predictors, potential confounders, and effect modifiers. Give diagnostic criteria, if applicable | Methods, Outcomes and Covariates paragraphs |
| Data sources/ measurement | 8* | For each variable of interest, give sources of data and details of methods of assessment (measurement). Describe comparability of assessment methods if there is more than one group | Methods, Data sources, Outcomes and Covariates paragraphs |
| Bias | 9 | Describe any efforts to address potential sources of bias | Not applicable |
| Study size | 10 | Explain how the study size was arrived at | All eligible participants were used, Methods, Study participants paragraph |
| Quantitative variables | 11 | Explain how quantitative variables were handled in the analyses. If applicable, describe which groupings were chosen and why | Methods, Covariates paragraph |
| Statistical methods | 12 | (*a*) Describe all statistical methods, including those used to control for confounding | Methods, Statistical analysis paragraphs |
|  |  | (*b*) Describe any methods used to examine subgroups and interactions | Not applicable |
|  |  | (*c*) Explain how missing data were addressed | Methods Statistical analysis paragraph 2 |
|  |  | (*d*) If applicable, explain how loss to follow-up was addressed | Not applicable, censoring described in Methods Study participants paragraph |
|  |  | (*e*) Describe any sensitivity analyses | Methods, Statistical analysis paragraph 2 |
| Results | | |  |
| Participants | 13* | (a) Report numbers of individuals at each stage of study—eg numbers potentially eligible, examined for eligibility, confirmed eligible, included in the study, completing follow-up, and analysed | Results paragraph 1 and Fig 1 |
|  |  | (b) Give reasons for non-participation at each stage | Results paragraph 1 and Fig 1 |
|  |  | (c) Consider use of a flow diagram | Fig 1 |
| Descriptive data | 14* | (a) Give characteristics of study participants (eg demographic, clinical, social) and information on exposures and potential confounders | Results paragraph 1 and Table 1 |
|  |  | (b) Indicate number of participants with missing data for each variable of interest | Results paragraph 1 and Table 1 |
|  |  | (c) Summarise follow-up time (eg, average and total amount) | Results paragraph 1 and Table 1 |
| Outcome data | 15* | Report numbers of outcome events or summary measures over time | Results paragraphs 1-3, Tables 1 and 2 |
| Main results | 16 | (*a*) Give unadjusted estimates and, if applicable, confounder-adjusted estimates and their precision (eg, 95% confidence interval). Make clear which confounders were adjusted for and why they were included | Table 3 and S3 Table |
|  |  | (*b*) Report category boundaries when continuous variables were categorized | Not applicable |
|  |  | (*c*) If relevant, consider translating estimates of relative risk into absolute risk for a meaningful time period | Not applicable |
| Other analyses | 17 | Report other analyses done—eg analyses of subgroups and interactions, and sensitivity analyses | Results Incidence rate ratios paragraph 4, S2 Table |
| Discussion | | |  |
| Key results | 18 | Summarise key results with reference to study objectives | Discussion, Summary of main results paragraph |
| Limitations | 19 | Discuss limitations of the study, taking into account sources of potential bias or imprecision. Discuss both direction and magnitude of any potential bias | Discussion, Limitations paragraphs |
| Interpretation | 20 | Give a cautious overall interpretation of results considering objectives, limitations, multiplicity of analyses, results from similar studies, and other relevant evidence | Discussion, Comparison with other studies paragraphs |
| Generalisability | 21 | Discuss the generalisability (external validity) of the study results | Discussion, Strengths and limitations paragraph 1 |
| Other information | | |  |
| Funding | 22 | Give the source of funding and the role of the funders for the present study and, if applicable, for the original study on which the present article is based | Funding paragraph |

**Note:** An Explanation and Elaboration article discusses each checklist item and gives methodological background and published examples of transparent reporting. The STROBE checklist is best used in conjunction with this article (freely available on the Web sites of PLoS Medicine at http://www.plosmedicine.org/, Annals of Internal Medicine at http://www.annals.org/, and Epidemiology at http://www.epidem.com/). Information on the STROBE Initiative is available at http://www.strobe-statement.org.
