# Supplementary figures and images for: Incidence and prevalence of primary care antidepressant prescribing in children and young people in England, 1998–2017: A population-based cohort study
Source: PLoS Med. 2020 Jul 22;17(7):e1003215. doi: 10.1371/journal.pmed.1003215 (PMC7375537; doi:10.1371/journal.pmed.1003215)

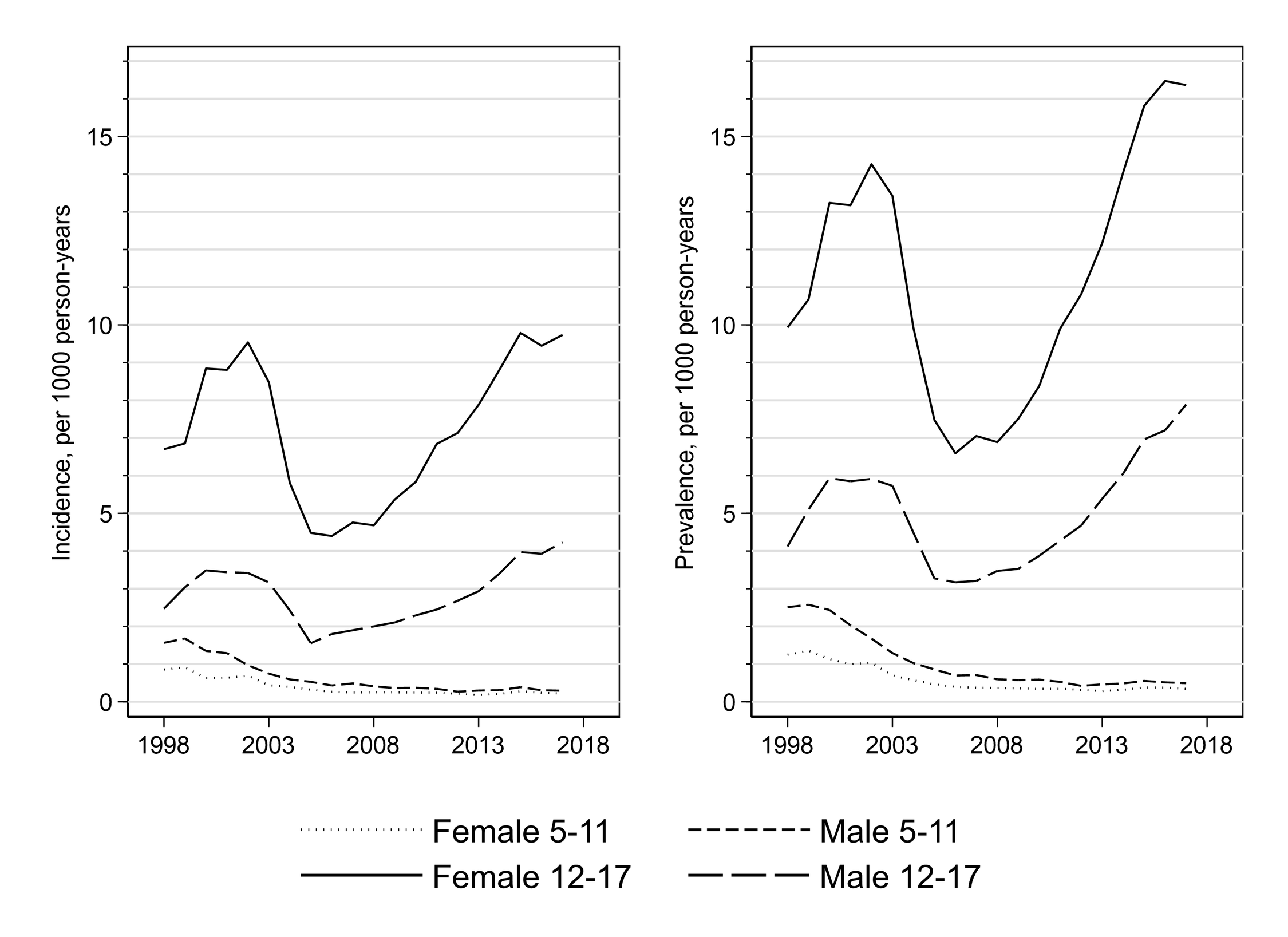

Supplement: S1 Fig — (TIF) [file pmed.1003215.s005.tif]

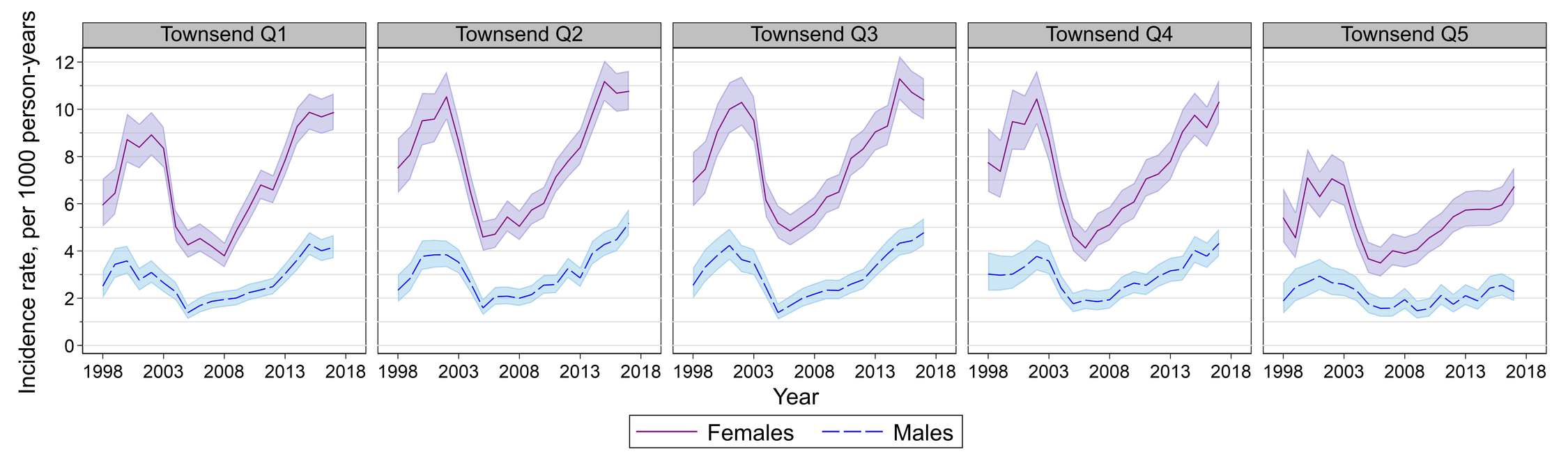

Supplement: S2 Fig — CI, confidence interval; Townsend Q1, least deprived quintile; Townsend Q5, most deprived quintile (TIF) [file pmed.1003215.s006.tif]

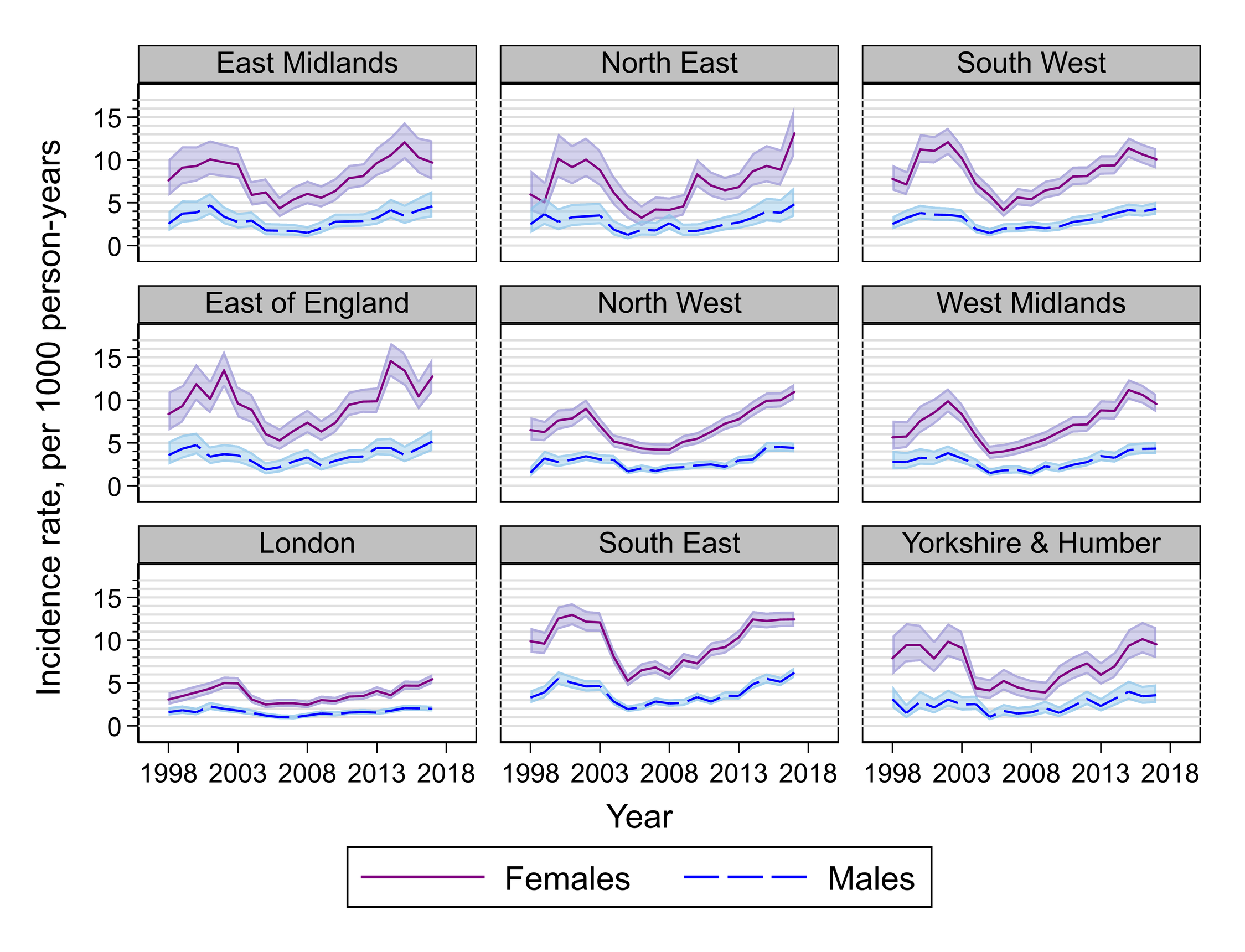

Supplement: S3 Fig — CI, confidence interval (TIF) [file pmed.1003215.s007.tif]

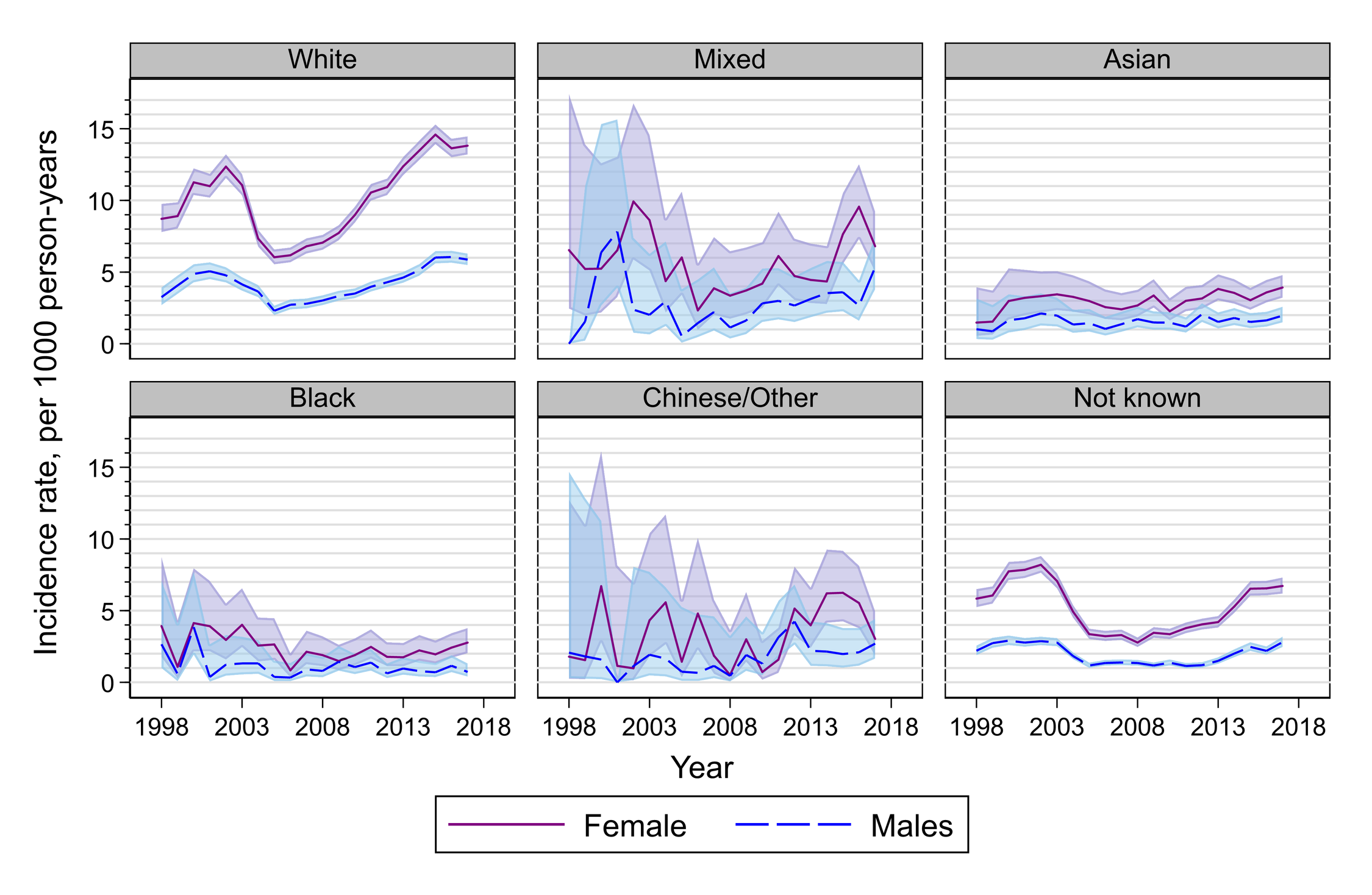

Supplement: S4 Fig — CI, confidence interval (TIF) [file pmed.1003215.s008.tif]

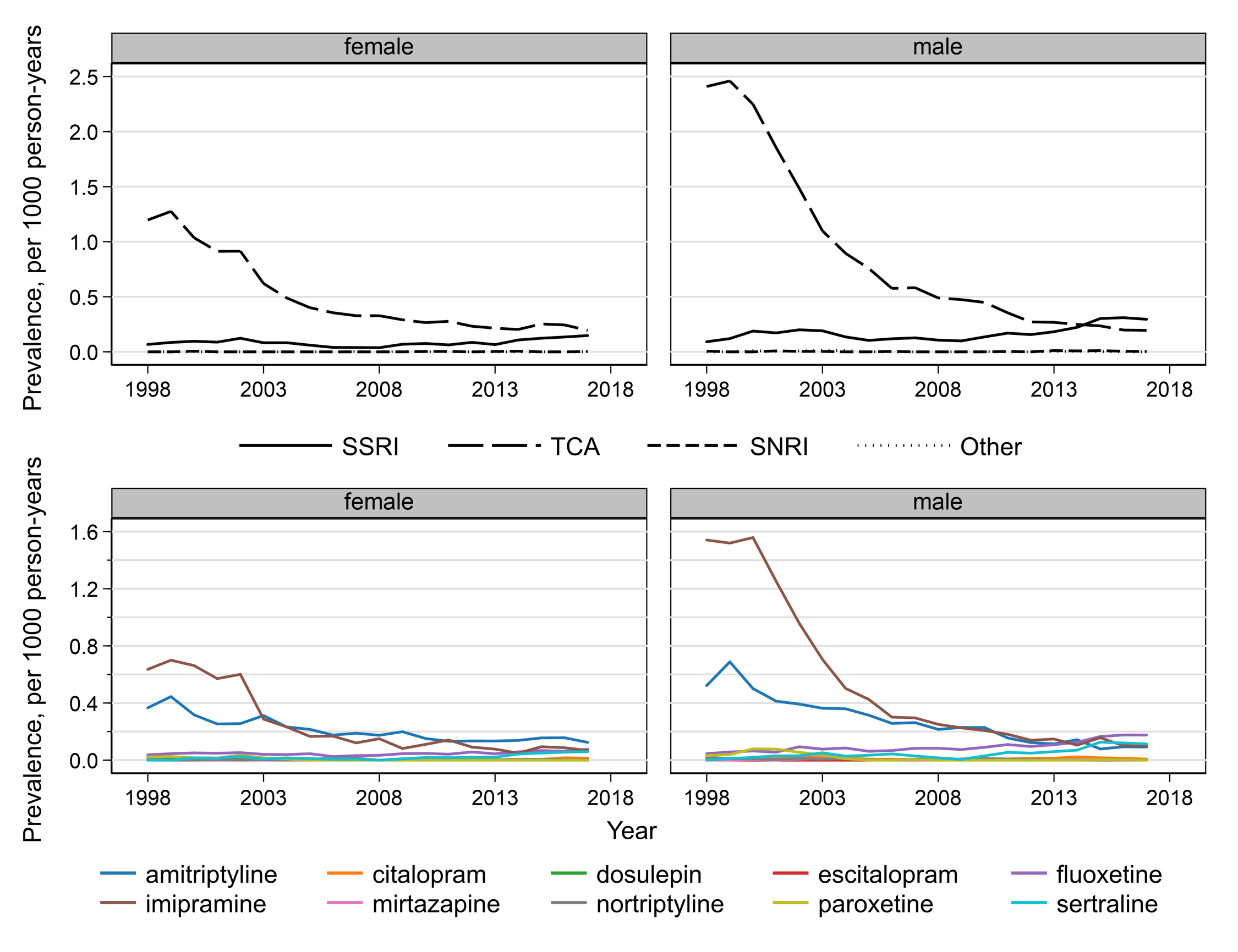

Supplement: S5 Fig — (TIF) [file pmed.1003215.s009.tif]

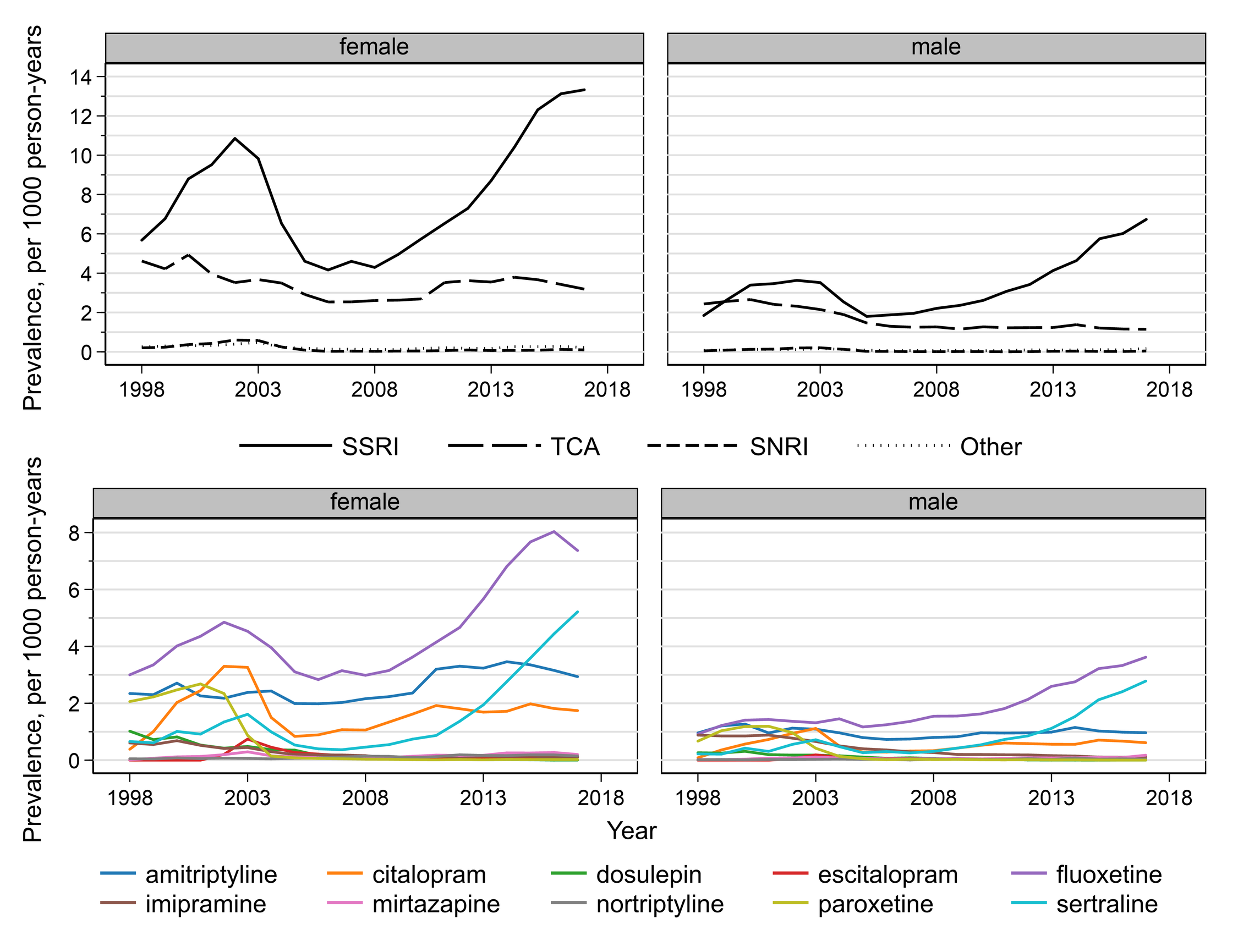

Supplement: S6 Fig — (TIF) [file pmed.1003215.s010.tif]

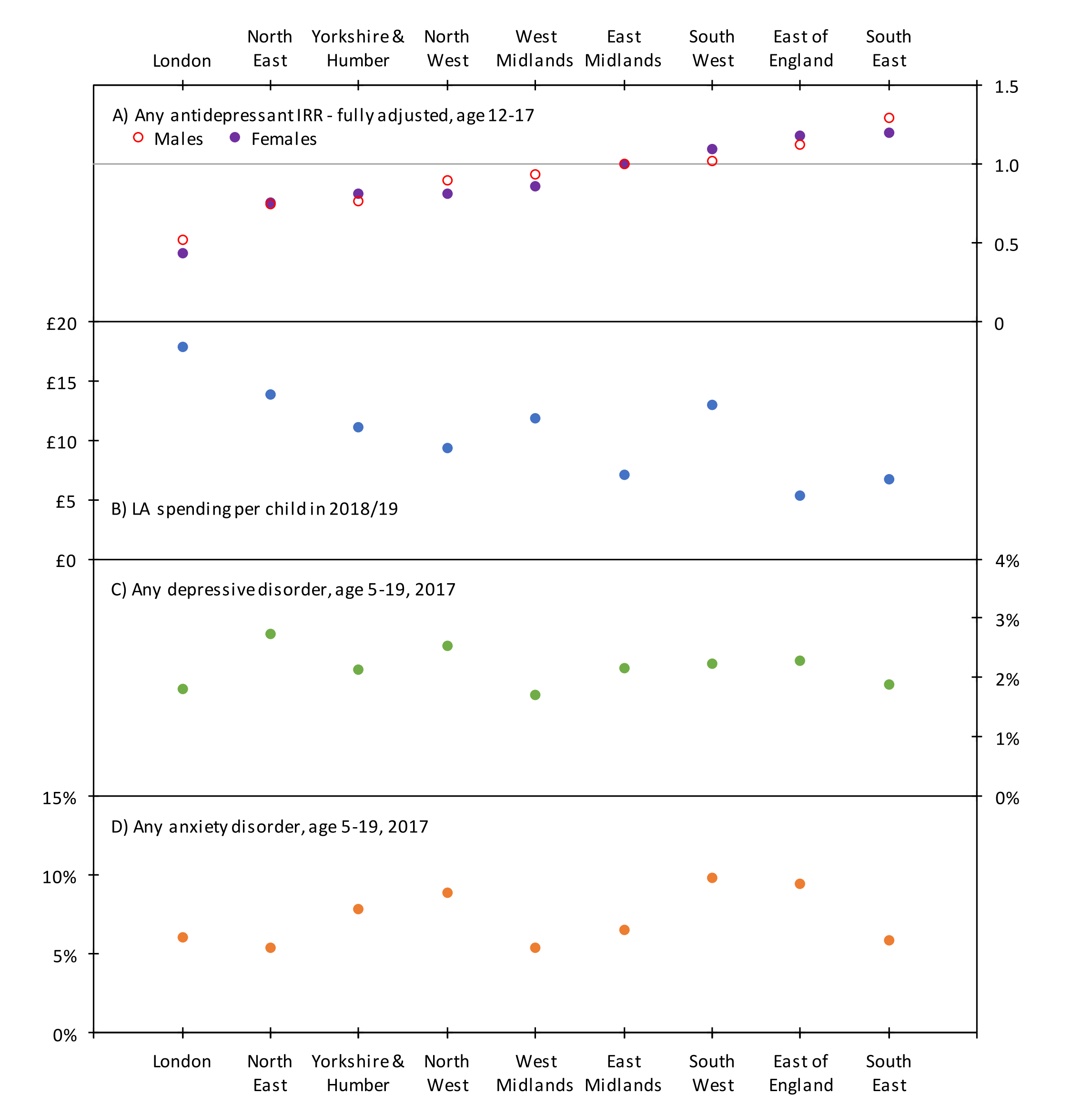

Supplement: S7 Fig — Regional (A) fully adjusted IRRs for any antidepressant, age 12–17 for males and females, 1998–2017; (B) LA spending on ‘low-level’ mental health services per child, 2018–2019 [30]; (C) prevalence of any depressive disorder, age 5–19, 2017 [6]; and (D) prevalence of any anxiety disorder, age 5–19, 2017 [6]. IRR, incidence rate ratio; LA, Local Authority. (TIF) [file pmed.1003215.s011.tif]
