# Supplementary material for: Incidence and prevalence of primary care antidepressant prescribing in children and young people in England, 1998–2017: A population-based cohort study
Source: PLoS Med. 2020 Jul 22;17(7):e1003215. doi: 10.1371/journal.pmed.1003215 (PMC7375537; doi:10.1371/journal.pmed.1003215)
Supplement: Attachment — Submitted filename: Plos Medicine Response to Reviewers.docx [file pmed.1003215.s012.docx]

Response to reviewers

We thank the editors and reviewers for their positive and helpful comments, and have detailed how we have addressed these below in blue. All line numbers refer to the marked version of the manuscript.  We hope that these changes sufficiently address the comments and that the manuscript is now suitable for publication.

Requests from the editors:

Noting PLOS' data policy, please adapt your data statement to briefly explain the criteria that will be applied to requests for access to study data, and include a non-author contact.

We have renamed and expanded the Data Availability Statement to include the following text [lines 506-511]:

Data Availability Statement: The patient level data from the QResearch database are specifically licensed according to its governance framework. Data access is limited to researchers who meet the eligibility criteria and have their project approved by the QResearch Scientific Committee. See <http://www.qresearch.org> for more details, including how to apply for data access.

Please begin the title with "Incidence and prevalence of ...", quote the study period, and add "in primary care" if appropriate.

The title has been updated as requested.

In your abstract, alongside the sizes of the cohorts studied, please quote aggregate participant characteristics from table 1.

We have added more characteristics information in the Abstract [lines 57-60].

Please add some additional quantitative details on study findings to your abstract, for example to support statements such as "... antidepressant prescribing decreased in 5- to 11-year olds".

We have added the incident antidepressant prescribing rates for 5- to 11-year-olds for the peak of 1999 and 2017, and the IRR and p-value for Black adolescents [lines 61-62, 67-68]. As the region and deprivation results apply to all age and sex groups, we feel adding in the figures would be confusing in the abstract.

To the "methods and findings" subsection of your abstract, please add a new final sentence quoting 2-3 of the study's main limitations.

We have added some of the study’s limitations to the Abstract [lines 69-71].

After the abstract, we will need to ask you to add a new and accessible "author summary" section in non-identical prose. You may find it helpful to consult one or two recent research papers published in PLOS Medicine to get a sense of the preferred style.

This has been added as requested [lines 83-104].

At line 368, please broaden the discussion of study limitations to cite relevant weaknesses of observational studies, for example.

We have added the limitation inherent in using secondary data of not having all the information we required to the Strengths and limitations section of the Discussion [line 435-436], and used the example of missing ethnicity information to highlight this.

Throughout the paper, please quote exact p values or p<0.001. Where available, please quote p values alongside 95% CI.

The p-values have been added as requested.

Please remove trademarks throughout the article.

The trademark in the Conflicts of interests [line 498] has been removed.

Please add institutional author names to references where appropriate, e.g., reference 1.

This has been added [line 520].

Please add accessed dates for online references.

These have been added [lines 527-635].

Please add a completed checklist for the most appropriate reporting guideline, which we imagine will be STROBE or RECORD, as a supplementary file (referred to in your methods section). In the checklist, please refer to individual items by section (e.g., "Methods") and paragraph number rather than by line or page numbers, as the latter generally change in the event of publication.

The STROBE checklist has been completed and uploaded.

Comments from the reviewers:

*** Reviewer #1:

I found this to be an interesting and well written article. The topic is of considerable public health importance and of wider societal concern, for the UK and for many other countries. The data source utilised is robust and the study design and statistical analyses seem appropriate. I have just a few minor critical comments, which are listed below.

1) It would be preferable to include at least one implication (for clinicians, public health experts, policymakers or researchers) in the 'Conclusions' subsection of the abstract.

We have expanded the Conclusions section of the abstract to include implications for future research around understanding the variation in antidepressant prescribing we found [lines 78-80].

2) The authors ought to say more about the risks associated with prescribing of amitriptyline and other tricyclic antidepressants given that: a) the drug is highly toxic in overdose, and the National Institute for Health and Care Excellence (NICE) recommended in November 2011 that the drug should not be prescribed to anyone with a history of self-harm (NICE, Clinical Guideline no. 133); b) incidence of self-harm has risen sharply among 13-16 year olds in the UK from 2011 (Morgan et al. BMJ 2017;359:j4351).

Thank you for this point. We have expanded this point and added in these references in the Implications section of the Discussion [lines 459-463]

3) In the Covariates subsection of the Methods, the authors make brief mention of missing data as regards the ethnicity variable that was available in the QResearch dataset. Further on in the manuscript, in the 'Strengths and limitations' subsection of the Discussion, information is given concerning the amount of data that are missing for this variable. It would be ideal if this more detailed information was provided in the Methods. Likewise, for any tables that report information pertaining to ethnicity, it would be useful for readers to be informed about the level of missing data for this variable via footnotes.

The overall number and percentage of those with ethnicity information not known is given in Table 1 for both the incidence cohort and those who had a new antidepressant prescribed during the study period. We have not included the breakdown for any characteristics in Table 3 with the incidence rate ratios as people can contribute person time in both age groups if not prescribed an antidepressant in the younger age group. The sensitivity analysis where we recoded those without ethnicity information to a new White / not known group is described in the statistical analysis section of the Methods [lines 215-217].

4) Given that they are geographically adjacent, it is an intriguing finding that the lowest prescription incidence rate observed was for London and the highest was for the South East. Many readers will likely be unaware that these two regions both lie in the South East of England, and so I suggest that this is explained.

Thank you for highlighting this result. London is located between the South East and East of England regions, the two areas which had the highest prescribing incidence rate ratio estimates for 12-17-year-olds. We have added this information to the Discussion [line 386-391].

*** Reviewer #2:

This is a pharmacoepidemiological study, using a general practice database in the UK (QResearch) to examine prevalence, incidence and associated temporal trends in antidepressant prescribing for 5-17 year olds.

A strength of the paper is its topical nature: data are available up to 2017. It has a large sample size and the sample is believed to be representative. While the analysis does not assess duration of therapy, it provides some relevant information on this topic since both incidence and prevalence estimates are presented. The trends over time are complex, and this is addressed with a piecewise regression approach in the multivariable component of the analysis.

The study is highly descriptive. Information on the reasons for prescribing are apparently not available, so the results (as the authors point out) are subject to various interpretations (e.g. see the non-specific interpretive statement at the start of the "implications" section. Nevertheless, it is important to understand patterns of drug use, and associated trends, even if this mainly generates hypotheses for additional studies. In addition to describing trends, model-based estimates are presented, which produce some interesting results.

The main findings are that there are differing trends in the 5-11 (decreasing) versus the 12-17 (increasing) age groups, and that the most commonly prescribed medications are those recommended by guidelines.

Another important finding is that the increase in antidepressants use in the 12-17 year olds has been substantial (a 2-3 fold increase between 2005 and 2017), which is important to know. Unfortunately, it is difficult to interpret since the absolute rates remain low, so the increase may either be a good or a bad thing. This is another example of where the study generates interesting hypotheses that should be address by additional research. Lower incidence in ethnic groups (Black and Asian) point towards possible issues of stigma and/or literacy in these groups.

Unadjusted estimates showed decreased prescribing in areas with increased deprivation whereas the opposite trend is seen in the adjusted analysis. Obviously some form of confounding (due to more ethnic minorities living in the most deprived areas?) is occurring but since the authors report only omnibus adjustments for multiple factors, it is not possible for readers to identify the source of this confounding.

Thank you for these comments. We have rewritten the deprivation paragraph in the Results section [lines 298-316] and added extra models showing the effect of adjusting for either region, ethnicity or year in S3 Table. These now show that, of these, only region is sufficient to reverse the association between prescribing and deprivation, and that after excluding London increased antidepressant prescribing was found with increasing deprivation in 12- to 17-year-olds and female 5- to 11-year-olds in the unadjusted analysis.

The authors make some interesting "ecological" comparisons, which leads to the generation of additional hypotheses. For, example, the possibility that low access to basic mental health care in regions that spend little on "low level" care, may lead to higher incidence of antidepressant use.

The incidence data is described as "first" use of an antidepressant, but the data collection appears to go only 12 months prior to cohort entry for some patients, so the study appears not to confirm first use.

We have clarified in the Outcomes section of the Methods that in some cases we had prescribing information from before the practice installed EMIS or a patient registered with a practice, due to electronic transferring of prescription data [lines 176-179].

In Table 3, the (trends within periods) rows doesn't seem to have a baseline group, so I'm not sure how to interpret the reported IRRs.

The IRRs for the different periods represent the linear trend within each period, i.e. the increase per year. We have added that the linear trend is “per year” to the Incidence rate ratios” section of the Results to make this point clearer [lines 291-294].

Overall, I feel that this is a good descriptive study that provides important information.

Thank you.

*** Reviewer #3:

Comments on ms PMEDICINE-D-20-00542

"Trends and variations in the incidence and prevalence of antidepressant prescribing in children and young people in England: a population-based cohort study

This article provides information about antidepressant prescription in the age group 5-17 yrs in England. The data source is a primary care database containing anonymised healthcare records from primary care, with an incidence cohort of 4.3 million children and young people.

Some comments:

The size of the database is impressive; however, I do not fully understand the degree to what this database covers the population of interest, i.e. children and adolescents <18 yrs of age with mental health problems/depression. The authors acknowledge that they do not have access to data from the specialist level mental health services, but could they describe how the health care system in the UK is organized? Where would a family turn to seek help for a depressed youth? How likely is it that your first visit would be in primary care? In many other countries, pharmacological treatment of depressed youth would be the sole responsibility of the specialist level services, and primary care would refer youth in need of pharmacological treatment to the specialist services. Is this different in the UK?

The cohort of interest is primarily the whole population of children and young people aged 5-17 years, as restricting the data to those with a coded record of depression or other mental health issues would mean we would exclude those where this information is missing, or have been prescribed antidepressants for other reasons, as described in the Strengths and limitations section of the Discussion [lines 431-433].

We have added in detail about the prescribing of antidepressants to children and young people after specialist assessment in the Introduction [line 128-131], and how in the UK GPs are the gatekeepers for children’s mental health issues and make decisions on specialist referral. GPs also typically are responsible for ongoing prescribing after a specialist child and adolescent mental health assessment. This is also described in the Strengths and limitations section of the Discussion [lines 424-427].

Without this information, it is impossible to assess the relevance of the results, both in regard to how generalizable they are to the entire UK population but also to other contexts outside the UK. A related question is if the geographical variation seen in the results could be explained by access to specialist level services (e.g. is that more accessible in the London area than in other parts of the UK)?

We have added that the vast majority of the UK population are registered with a GP practice (as described by Vezyridis & Timmons, 2016), and QResearch has been shown to be representative of the population to the Discussion Strengths and limitations [lines 415-417].

While we cannot directly assess the relationship between access to specialist services and prescribing from the data we have, this is an interesting idea that should be explored further. We have attempted to assess the broad association between spending on early interventions and antidepressant prescribing in children and young people in S7 Figure and the Results [lines 348-350], and suggested that this is an important area for future research in the Implications section of the Discussion [line 469-472].

I ran across an article on the UK primary care database (Moore ea, BMJ 2009) that may be of relevance. <https://www.bmj.com/content/bmj/339/bmj.b3999.full.pdf>

Thank you for identifying this reference. As this study does not look at prescribing patterns in under 18s separately, we have chosen not to add this reference.

The authors have added a reference in the introduction that shows that the waitlist to see a mental health specialist was long in 2017. How would a "long wait-list condition" affect patterns of seeking help? Would it be likely that youth instead contacted primary care, or would it lead to a delay in treatment?

We have highlighted that GPs are the first point of contact and that they are supposed to make a referral, when appropriate as per current guidelines, to a specialist in child and adolescent mental health (Introduction lines 128-131 and Discussion, lines 424-427). Long waits for these specialists may therefore drive increased prescribing in primary care for moderate to severe depression in young people without an initial specialist mental health assessment.

In this regard, please explain what "low-level services" mean and, if possible, how the organisation of the service system may have influenced the results (or how we can understand them). Does a "low-level service" provide only psychosocial interventions, or do they also prescribe meds? Would they refer a youth in need of meds to some other health care level, and if so, which one? The authors state (line 327-328) that the absence of psychosocial support could lead to an increased prescribing of antidepressants. It could actually be the other way around; with increased access to psychosocial counselling, more youth would meet someone who could identify the need for pharmacological treatment.

We have expanded the definition of “low-level mental health services” in the Methods [lines 222-223] to “those which are non-specialist, preventative and early intervention, which fall below specialist referral thresholds”. This could include GPs who may prescribe medication, although initiation of prescriptions should be done after consultation with a specialist.

Given the unclarity of the extent to which the included cohort represents the general population, think the title may be misleading. It is stated that this is a "population-based cohort study" - but if only part of the population is included in the study that must be clearly stated.

While QResearch doesn’t cover all primary care providers in England, it has been shown to be the most representative primary care database available in the UK, as described in the Strengths and limitations section of the Discussion [lines 416-417] based on the work by Kontopantelis et al. (2018, BMJ Open).

The paper would improve by including perspectives from other countries in Europe and elsewhere, e.g Abbin-Karahagopian, Herta ea (2014, Eur J Clin Pharmacol), Noordam ea (2015, Eur J Clin Pharmacol), Lagerberg ea (2018, J European J of Child and Adolescent Psychiatry).

Thank you for identifying these extra references. We have included Lagerberg et al in the Introduction [line 137]. However the other references had limited information specifically on children and young people. We have tried to keep the focus on this younger age group and have therefore decided not to add these references.

The stated aims were to describe trends, estimate variation in uptake, and assess to what extent the NICE guidelines were adhered to. I am not sure what "variation in uptake" actually means in this study, especially not knowing to what extent this database covers the entire population.

QResearch has been shown to be the most representative primary care database available, as described in the Strengths and limitations section of the Discussion [lines 416-417] based on the work by Kontopantelis et al. (2018, BMJ Open). We have changed the wording of objective 2 in the Introduction to be “variation in prescribing” to make be clearer [line 154].

Please explain the term "Townsend deprivation quintiles".

We have added more details and a reference to the Townsend deprivation index in the Covariates section of the Methods [lines 193-197]

*** Reviewer #4:

This is an interesting and useful study on the trends and variation in the incidence and prevalence of antidepressant prescribing in children and young people in England. The study design, datasets, statistical methods and analyses, and presentation (tables ang figures) and interpretation of the results are mostly adequate. However, there are still a few important issues needing attention.

1) Strictly speaking, it's a cohort study. As it's not about incidence/prevalence in the population, it's better not to use 'population-based' in case mis-understanding and mis-interpretation.

As we have examined antidepressant prescribing in the population without restriction, we deem that “population-based cohort study” is an accurate description. This usage is also in line with articles previously published in PLoS Medicine (e.g. Brauer R et al (2019) Trazodone use and risk of dementia: A population-based cohort study PLoS Med. 2019 Feb 5;16(2):e1002728; Lin KJ et al (2020) Prescribing systemic steroids for acute respiratory tract infections in United States outpatient settings: A nationwide population-based cohort study. PLoS Med 17(3): e1003058; Fralick M et al (2019) Desmopressin and the risk of hyponatremia: A population-based cohort study PLoS Med 16(10): e1002930).

2) In the conclusion of the abstract, it says 'the trends and variation in antidepressant prescribing found may reflect true differences in need and risk factors, access to diagnosis and services, prescribing behaviour, or young people's help-seeking behaviour'. However, apart from analyses of trends in incidence and prevalence of antidepressant prescriptions over time, there are no data or formal analyses on all those explanatory factors listed in the conclusion such as risk factors, access to services, prescribing behaviours and etc. It's good that the authors have done a nice job on the trends of prescription of antidepressant, but in the term of comprehensively explaining the trends, it seems a bit lacking solid evidence and analyses. Can authors find out the data within the cohorts on underlying conditions/co-morbidities, and their severity, related hospital care, and other risk factors, and carry out analyses to show the trends so that can offer some explanations on trends of prescriptions?

The conclusion of the abstract was an attempt to suggest future areas of research. As we state in the Implications section of the Discussion [lines 444-447] the study was not designed to determine the reasons behind the antidepressant prescribing patterns and variation we found. We have for the first time shown variation in antidepressant prescribing in children and young people in different areas of England, which may represent differences due to need or inequity due to other factors that we have not investigated here. We welcome future research that can lead to a better understanding of these underlying causes.

3) Figure 2 and 3 are useful. However, are they adjusted incidence rates? If not, can we see the adjusted incidence rates in one way or another (comprehensively adjusted for everything)?

4) Table 3 on IRRs are mostly fine. However, when talking about fully adjusted IRRs, it became a bit limited as only adjusted for age, sex, deprivation, region, and ethnicity which are mostly demographics. The other important factors such as diagnosis, case-mix, hospital care, and other risk factor are not adjusted. If the data are not available, then at least should address this carefully in the limitations.

Thank you for both of these points about adjustment. We have provided crude incidence rates separately by sex and age group in Figs 2 and 3, to show the actual prescribing patterns over time. We then provide adjusted incidence rate ratios in Table 3. For this analysis, we limited our analysis to adjusting for demographic information only to identify the broader patterns of antidepressant prescribing. Our separate paper examining secondary care specialist visits and indications recorded around the earliest primary care antidepressant prescription goes into more detail about these elements (Jack RH et al *BMC Medicine* 2020;18(1):93. doi: 10.1186/s12916-020-01560-7).
