# Supplementary material for: Incidence and prevalence of primary care antidepressant prescribing in children and young people in England, 1998–2017: A population-based cohort study
Source: PLoS Med. 2020 Jul 22;17(7):e1003215. doi: 10.1371/journal.pmed.1003215 (PMC7375537; doi:10.1371/journal.pmed.1003215)
Supplement: Attachment — Submitted filename: Plos Medicine Response to Reviewers2.docx [file pmed.1003215.s013.docx]

Response to reviewers

We were pleased that our previous version had sufficiently addressed the points raised by Reviewers 1 and 4. We would like to thank the editors and Reviewer 3 for their additional comments, to ensure the manuscript is clear and detailed. We have described how we have addressed these comments below in blue. All line numbers refer to the tracked version of the manuscript, which also contains production changes suggested in a separate email.  We hope that these changes sufficiently address the comments and that the manuscript is now suitable for publication.

Requests from Editors:

In your data statement (in the submission form, to appear in the article metadata upon publication) we suggest substituting the following point of access: <https://www.qresearch.org/information/information-for-researchers/>.

This has been amended in the online form.

We suggest quoting 1-2 additional quantitative findings in your abstract - for example, the finding quoted at line 329 would seem of interest.

We have added the results about the strongest trend in prescribing by deprivation in 12-17-year-old females in the uploaded manuscript. However, due to the other additional changes listed below, this meant the abstract was over 500 words, and could not be uploaded in the online manuscript data. We have therefore excluded this sentence from this separate version.

Also, we would suggest adding a sentence, say, to the abstract to quote quantitative observations on the classes of antidepressants prescribed.

We have added the findings about the first prescribed class of antidepressants for SSRIs and TCAs.

Please adapt the abstract so that 95% CI are quoted along with incidence rate ratios and p values.

We have included the 95% CIs for the incidence rate ratios in the abstract.

In the sentence addressing study limitations at the end of the "methods and findings" subsection of your abstract, please quote one further limitation - possibilities could be that antidepressant use has not been measured, and the issue of unmeasured confounding. We would also suggest including these issues in the element of the discussion section summarizing study limitations.

We have included in the Abstract and Strengths and Limitations section of the Discussion that we were only able to assess whether antidepressants had been prescribed, and not whether they had been dispensed or taken, and added in the Discussion that there may still be unmeasured confounding.

You mention that the study protocol has been published (line 173). Please highlight analyses that were not prespecified.

We have moved the reference to the study protocol to the start of the Methods section, and added in how the analyses that were not prespecified are described:

“Any analyses that were not prespecified in the protocol are described as sensitivity or post-hoc analyses below.”

Please refer to the attached STROBE checklist in your methods section (e.g., "See S1_STROBE").

A sentence referring to the STROBE checklist has been added to the start of the Methods section.

Please move the ethics statement from the end of the ms to the methods section.

This section has been moved.

Noting instances of "p<0.0001" in the tables, please quote exact p values or "p<0.001" throughout, unless there are specific statistical reasons to do otherwise.

All p values in the tables have been updated to be to 3 decimal places, including those which are less than 0.001.

Please remove the information on funding, competing interests and data availability from the end of the ms - this information will appear in the article metadata (via the submission form).

These sections have been removed.

Please make that "PLoS ONE" in the reference list

This has been updated.

Comments from Reviewers:

*** Reviewer #1:

The authors have adequately addressed all of my comments.

*** Reviewer #3:

Comments on ms PMEDICINE-D-20-00542R2

"Trends and variations in the incidence and prevalence of antidepressant prescribing in children and young people in England: a population-based cohort study

The manuscript has been improved in several ways.

The authors provide more information on the clinical practice in England with regard to CYP with mental health problems.

They have also provided more information on the QResearch database and how they have tried to link the results to other data (i.e. the Townsend deprivation index).

The strength of this study is the size of the cohort and the long time period studied (which the authors themselves have identified, line 455-466). The identified trends are similar to what is seen in many other countries. The authors also do a good job in trying to relate their findings to other data of interest (demographic variations etc).

Since the aim is primarily descriptive, it is a weakness (also identified) that there is no data provided on what is prescribed from the specialist level, and lack of information on diagnoses/indications. It is stated that "In the UK, prescribing antidepressants to CYP should only be done after assessment and diagnosis by a child and adolescent psychiatrist or other expertise in child and adolescent mental health". To me, this seems as a quite strong recommendation and most likely, the results would be affected if specialist level data was provided alongside what is prescribed within on primary care level.

To try and be clear that the statement quoted is about guidelines, we have added “According to NICE guidelines” at the beginning.

I agree with reviewer #4 that this is should be viewed as a cohort study; it could actually strengthen the argumentation. The authors provide support for the use of the QResearch database, and its representativity of the entire population. I accept that - but still we can assume that a lot of information on the issue in focus - prescription of antidepressants to CYP- are not included in this dataset.

We are confident that we are capturing all the antidepressant prescriptions for the study population that were prescribed within primary care.

Given that the recommendation is that antidepressants should be described by specialists, the authors may claim that they with this impressive data set can show that this is not the case. They do have strong evidence that there is a large number of prescriptions of antidepressants being done within the primary care health services, with regional and other variations. Given that the specialist mental health services should be responsible for prescription of antidepressants one would assume that there should be very little (or no) prescriptions, or that the prescription patterns within primary care should be affected by change in policy or access to mental health services. The last question is also what the authors have tried to study when comparing regional variations in prescribing with other data on mental health resources.

One aim of the study was to assess to what extent the NICE guidelines for CYP are being adhered to. Is it possible to claim that based on these findings, guidelines are generally adhered to with regard to which antidepressants are prescribed, but not the way in which they are prescribed? I understand that GPs many times may need to deal with problems that "should" be handled on the specialist level, and that this pragmatic way of taking care of a medical problem may work out just fine and may be better than doing nothing. Still, these data provide a matrix for discussing the strengths and weaknesses in how youth get access to care.

I think the study has its merits, but primarily in the sense described above. It does not give a full picture of prescription patterns in England but rather a full picture of prescription patterns within primary care in England. The title should be adjusted accordingly.

The title of the study refers to “primary care antidepressant prescribing”.

*** Reviewer #4:

I am satisfied with the authors' response. No further issues needing attention.

***
